# pH and thiosulfate dependent microbial sulfur oxidation strategies across diverse environments

**DOI:** 10.3389/fmicb.2024.1426584

**Published:** 2024-07-19

**Authors:** Lauren E. Twible, Kelly Whaley-Martin, Lin-Xing Chen, Tara Colenbrander Nelson, James L.S. Arrey, Chad V. Jarolimek, Josh J. King, Lisa Ramilo, Helga Sonnenberg, Jillian F. Banfield, Simon C. Apte, Lesley A. Warren

**Affiliations:** ^1^Department of Civil and Mineral Engineering, University of Toronto, Toronto, ON, Canada; ^2^Department of Earth and Planetary Science, University of California, Berkeley, Berkeley, CA, United States; ^3^School of Mathematical and Physical Sciences, University of Technology Sydney, Ultimo, NSW, Australia; ^4^Commonwealth Scientific Industrial and Research Organization, Black Mountain, ACT, Australia; ^5^EcoReg Solutions, Guelph, ON, Canada; ^6^Commonwealth Scientific Industrial and Research Organization, Clayton, VIC, Australia

**Keywords:** sulfur oxidizing bacteria (SOB), pH, tailings impoundments, sox genes, thiosulfate

## Abstract

Sulfur oxidizing bacteria (SOB) play a key role in sulfur cycling in mine tailings impoundment (TI) waters, where sulfur concentrations are typically high. However, our understanding of SOB sulfur cycling via potential S oxidation pathways (*sox*, r*dsr*, and S_4_I) in these globally ubiquitous contexts, remains limited. Here, we identified TI water column SOB community composition, metagenomics derived metabolic repertoires, physicochemistry, and aqueous sulfur concentration and speciation in four Canadian base metal mine, circumneutral-alkaline TIs over four years (2016 – 2019). Identification and examination of genomes from nine SOB genera occurring in these TI waters revealed two pH partitioned, metabolically distinct groups, which differentially influenced acid generation and sulfur speciation. Complete *sox* (c*sox*) dominant SOB (e.g., *Halothiobacillus* spp., *Thiomonas* spp.) drove acidity generation and S_2_O_3_^2-^ consumption via the c*sox* pathway at lower pH (pH ~5 to ~6.5). At circumneutral pH conditions (pH ~6.5 to ~8.5), the presence of non-c*sox* dominant SOB (hosting the incomplete *sox*, r*dsr*, and/or other S oxidation reactions; e.g. *Thiobacillus* spp., *Sulfuriferula* spp.) were associated with higher [S_2_O_3_^2-^] and limited acidity generation. The S_4_I pathway part 1 (*tsdA*; S_2_O_3_^2-^ to S_4_O_6_^2-^), was not constrained by pH, while S4I pathway part 2 (S_4_O_6_^2-^ disproportionation via *tetH*) was limited to *Thiobacillus* spp. and thus circumneutral pH values. Comparative analysis of low, natural (e.g., hydrothermal vents and sulfur hot springs) and high (e.g., Zn, Cu, Pb/Zn, and Ni tailings) sulfur systems literature data with these TI results, reveals a distinct TI SOB mining microbiome, characterized by elevated abundances of c*sox* dominant SOB, likely sustained by continuous replenishment of sulfur species through tailings or mining impacted water additions. Our results indicate that under the primarily oxic conditions in these systems, S_2_O_3_^2-^ availability plays a key role in determining the dominant sulfur oxidation pathways and associated geochemical and physicochemical outcomes, highlighting the potential for biological management of mining impacted waters via pH and [S_2_O_3_^2-^] manipulation.

## 1 Introduction

Biological sulfur oxidation can present significant risks to the environment via acid generation, contaminant mobilization, and oxygen consumption (i.e., acid mine drainage) (Edwards et al., 1999; Elberling and Damgaard, 2001; Baker and Banfield, 2003; Druschel et al., 2003; Johnson and Hallberg, 2003; Korehi et al., 2014). This biological acidity production occurs in both natural (termed acid rock drainage, found in alpine catchments; Lacelle et al., 2007; Zarroca et al., 2021) and anthropogenic (e.g., mine tailings impoundments and waste rock piles; Akcil and Koldas, 2006) environments, though the scale to which it occurs in anthropogenic environments is typically much larger.

Base metal mine tailings impoundment (TI) wastewaters often have elevated sulfur concentrations due to the dominance of sulfide minerals in base metal ores [e.g., chalcopyrite (CuFeS_2_), sphalerite ((Zn, Fe)S), etc.]. Sulfides can be partially oxidized during the grinding, flotation, and leaching steps of sulfide mineral ore extraction (Liljeqvist et al., 2011), resulting in the production and subsequent release of sulfur oxidation intermediate compounds (SOI) from tailings streams to TIs. In addition to sulfide (ΣH_2_S), SOI commonly found in mining impacted waters include thiosulfate (S_2_O_3_^2-^), tetrathionate (S_4_O_6_^2-^), elemental sulfur (S^0^), sulfite (SO_3_^2-^), and a range of other polythionates (S_x_O_y_^2-^) (Makhija and Hitchen, 1979; Miranda-Trevino et al., 2013; Whaley-Martin et al., 2020). The concentrations of individual SOI in TI wastewater can vary significantly spatially, seasonally, and amongst mining operations. SOI may be present in relatively high concentrations in tailings slurries, but are typically much lower in the TI waters due to dilution by other water inputs collected in these actively managed systems (Thamdrup et al., 1994), though they are typically higher than concentrations observed in natural environments (Foucher et al., 2001; Canfield and Farquhar, 2009; Silva et al., 2012; Camacho et al., 2020b; Vincent et al., 2021). SOI can be reduced, oxidized, and disproportionated, resulting in differential SOI speciation and pH outcomes, via both abiotic and biotic reactions (Philippot et al., 2007; Klatt and Polerecky, 2015; Houghton et al., 2016) further contributing to the complexity of the sulfur cycle in these environments. As mine TI systems continue to grow in number and size around the world, an understanding of the biogeochemical cycling of sulfur compounds occurring within these contexts, the SOB involved, and the influencing factors determining water quality outcomes is increasingly important.

Few studies have addressed the coupling of sulfur oxidation metabolic pathways in TIs to SOB taxonomy, physicochemistry, and sulfur geochemistry (Whaley-Martin et al., 2019, 2023; Miettinen et al., 2021), contrasting more well-studied extremophilic acid mine drainage environments (Bond et al., 2000; Johnson and Hallberg, 2003; Dold, 2014). Recent research has highlighted a divergence of microbial communities found in circumneutral mining impacted TI waters from those of acid mine drainage environments (Whaley-Martin et al., 2019, 2023; Camacho et al., 2020b; Lopes et al., 2020; Miettinen et al., 2021). A number of sulfur oxidation pathways have been identified as being used by SOB potentially found in mining environments, though the physicochemical, geochemical, and/or ecological parameters governing which S oxidation pathway(s) occur are not well defined (Han and Perner, 2015; Wang et al., 2016; Watanabe et al., 2019). These pathways include the sulfur oxidation (*sox*), reverse dissimilatory sulfite reductase (r*dsr*), and tetrathionate intermediate (S_4_I) pathways (Friedrich et al., 2001; Han and Perner, 2015; Klatt and Polerecky, 2015; Wang et al., 2016; Watanabe et al., 2019; Whaley-Martin et al., 2023). The *sox* pathway has seven structural genes which encode four proteins (soxXA, soxYZ, soxB, and soxCD) allowing this pathway to mediate S_2_O_3_^2-^, SO_3_^2-^, S^0^, and ΣH_2_S dependent cytochrome c reduction (Friedrich et al., 2001). SoxAX catalyzes the attachment of S_2_O_3_^2-^ to cysteine residue on the soxY of the soxYZ complex (forming soxZY-Cys-S^-^) (Friedrich et al., 2000, 2001; Bamford et al., 2002; Watanabe et al., 2019). The soxCD complex then oxidizes the sulfate sulfur from SoxZY-Cys-S^-^ to produce the sulfonate group as soxZY-Cys-SO_3_^-^ (Quentmeier et al., 2000; Zander et al., 2011; Watanabe et al., 2019). SoxB further hydrolyzes the sulfane sulfur from SoxZY-Cys-SO_3_^-^ to a free sulfate ion (Quentmeier et al., 2000; Zander et al., 2011; Watanabe et al., 2019). Free SOI are not produced by the *sox* pathway when found in its complete form (complete *sox*; c*sox*) as they are covalently attached to *soxYZ* until complete oxidation to sulfate (SO_4_^2-^; Grabarczyk and Berks, 2017). An incomplete form of the *sox* pathway (lacking *soxCD,* i*sox*) has also been identified which can form S^0^ (Frigaard and Dahl, 2008; Watanabe et al., 2019). This S^0^ can be subsequently oxidized by r*dsr,* sulfur-oxidizing heterodisulfide reductase-like (s*hdr*), or sulfur dioxygenase (*sdo*) to SO_3_^2-^ (Klatt and Polerecky, 2015). The r*dsr* pathway is composed of the same proteins as the *dsr* pathway (*sat*, *aprAB*, *dsrAB*) though the reductive and oxidative varieties are phylogenetically discernible (van Vliet et al., 2021). SOI including S_2_O_3_^2-^, S^0^, and ΣH_2_S, can be oxidized through the r*dsr* pathway and generate free SO_3_^2-^ (Klatt and Polerecky, 2015). Sulfane sulfur generated from the i*sox* pathway can then be transported to the cytoplasm in the form of persulfides (R-S-S^-^) to be further oxidized to HSO_3_^-^ by *dsrAB* or s*hdr* (Pott and Dahl, 1998; Frigaard and Dahl, 2008; Cao et al., 2018; Koch and Dahl, 2018). The HSO_3_^-^ can be further oxidized to APS (adenosine 5’-phosphosulfate) by *aprBA* with electron transfer by *aprM* or *hdrAACB* and then to SO_4_^2-^ by *sat* (Meyer and Kuever, 2007; Loy et al., 2009). R*dsr* pathway presence is typically associated with high energy efficiency compared to the *sox* pathway (Klatt and Polerecky, 2015). Several beta- and gammaproteobacteria have been found to utilize the S_4_I pathway (or Kelly-Trudinger pathway) which generates free S_4_O_6_^2-^ (Dam et al., 2007). The S_4_I pathway oxidizes S_2_O_3_^2-^ to S_4_O_6_^2-^ by thiosulfate dehydrogenase (*tsdA*) or thiosulfate:quinol oxioreductase (*TQO* or *doxD*; Brito et al., 2015; Wang et al., 2016; Hutt et al., 2017). Subsequent processing of free S_4_O_6_^2-^ can be catalyzed by tetrathionate reductase (*ttrABC*) to produce S_2_O_3_^2-^ or tetrathionate hydrolase (*tetH*) to produce S_2_O_3_^2-^, S^0^, and SO_4_^2-^, which are both common in SOB (Wang et al., 2016; Camacho et al., 2020a; Miettinen et al., 2021). A recent study (Whaley-Martin et al., 2023) identified oxygen as a control on whether the c*sox* (high O_2_) or r*dsr* (low O_2_) pathway dominated in one TI with differing water quality outcomes, indicating that physicochemical and/or SOI substrate partitioning of SOB within TI may happen more broadly.

The objectives of this cross-mine study were to identify mining TI associated SOB, examine functional differences in SOB communities, and align their associated S oxidizing repertoires to geochemical and physicochemical characteristics and outcomes in circumneutral TI waters of four base metal mines located across Canada (Manitoba, Newfoundland, Ontario). A better understanding of these genetic, geochemical, and/or physicochemical connections will inform biological management strategies as well as further understanding of S biogeochemical cycling more broadly. Four years (2016 – 2019) of S geochemistry, physicochemistry, and genus level community structure and function data from the four base metal mine TIs were examined. To determine if the findings of this study were site specific or reflective of broader environmental trends, comparisons were made to published studies on other mines and industrial environments, as well as natural environments.

## 2 Materials and methods

### 2.1 Site descriptions

Four base metal mine TI waters were sampled from 2016 to 2019 resulting in a total of 42 water samples (see Supplementary Figure S1). These four mines are located across central and eastern Canada and consist of: Mine 1 in Flin Flon, Manitoba (Cu, Zn, Au, Ag), Mine 2 in Sudbury, Ontario (Ni, Cu, Co, Pt, Pd), Mine 3 in Snow Lake, Manitoba (Cu, Zn, Se, Te, Ag), and Mine 4 in Baie Verte, Newfoundland (Cu, Au). The mines range in size, age, and stage of development with Mine 1 and Mine 2 being the oldest (operating on and off since 1927 and 1928, respectively), Mine 3 opened in 1979 and Mine 4 as the youngest with mining originating on the property around 1997, with the commissioning of the TI in 2009 (Table 1). The TI facilities across sites also vary in size and depth with Mine 2 being both the largest and deepest at ~38 m, followed by Mine 1 at ~7 to 10 m depth and finally Mine 3 and Mine 4 at ~1.5 to 2.5 m depth. TI water cover sampling depths for Mine 1, Mine 2, Mine 3, and Mine 4 ranged 5 to 10 m, 0.5 to 10 m, 0.5 to 3 m, and 0.5 to 1.5 m, respectively, and this investigation targeted seasonal open water collection (early spring to late fall).

**Table 1:** Mine information, sulfur geochemistry, and physicochemistry data for Mine 1, Mine 2, Mine 3, and Mine 4 tailings impoundment waters (2016 – 2019) presented as average (± standard deviation).

	Mine 1	Mine 2	Mine 3	Mine 4
Year established	1927	1928	1979	2009
Target elements	Cu, Zn, Au, Ag	Ni, Cu, Co, Pt, Pd	Cu, Zn, Se, Te, Ag	Au, Cu
Facility maximum depth (m)	~10	~38	~2.5 – 3	~1.5
Number of samples (n)	13	15	6	8
Total sulfur 0.45 μm(mmol/L)	11.3 – 16.8(avg. = 14 ± 1.6)	7.5 – 9.8(avg. = 8.9 ± 0.7)	7.0 – 9.6(avg. = 7.9 ± 1.0)	0.3 – 2.6(avg. = 1.9 ± 0.8)
	S_React_(mmol/L)	0.5 – 9.8(avg. = 3.9 ± 2.3)	0.0 – 2.3(avg. = 1.1 ± 0.8)	0.8 – 2.4(avg. = 1.3 ± 0.5)	0.0 – 1.0(avg. = 0.5 ± 0.3)
S-S_2_O_3_^2-^(mmol/L)	0.00 – 1.20(avg. = 0.73 ± 0.36)	0.02 – 0.48(avg. = 0.18 ± 0.15)	0.00 – 0.10(avg. = 0.04 ± 0.04)	0.00 – 0.10(avg. = 0.02 ± 0.03)
S-SO_3_^2-^(mmol/L)	0.00 – 0.21(avg. = 0.03 ± 0.05)	0.00 – 0.05(avg. = 0.02 ± 0.01)	0.00 – 0.06(avg. = 0.02 ± 0.02)	0.00 – 0.10(avg. = 0.01 ± 0.03)
S-SO_4_^2-^(mmol/L)	4.0 – 13.2(avg. = 9.8 ± 2.4)	6.1 – 10.2(avg. = 8.1 ± 1.3)	6.0 – 7.4(avg. = 6.6 ± 0.6)	0.3 – 2.2(avg. = 1.4 ± 0.6)
pH	5.5 – 11.8(avg. = 9.7 ± 2.0)	5.1 – 8.3(avg. = 6.3 ± 0.9)	6.6 – 7.6(avg. = 7.1 ± 0.4)	7.2 – 9.0(avg. = 8.0 ± 0.7)
Dissolved Oxygen(%)	1.8 – 88.6(avg. = 44.6 ± 25.7)	0.5 – 112(avg. = 36.2 ± 30.0)	1.3 – 93.8(avg. = 54.5 ± 33.6)	66.8 – 95.0(avg.* = 82.6 ± 8.8)
Temperature(°C)	2.7 – 16.1(avg. = 6.7 ± 4.1)	2.8 – 22.5(avg. = 10.9 ± 5.2)		3.2 – 18.9(avg.* = 8.8 ± 5.7)	2.0 – 21.6(avg. = 8.2 ± 5.9)

^*^Missing data point (n-1).

### 2.2 Physicochemical characterization and sampling scheme

Samples collected from Mine 2 TI were taken from two different points in the reservoir (see Supplementary Figure S2) which include a floating platform at the deepest point of the reservoir and the outflow dam. The outflow dam represents an approximate average of the overall water column and is ~2 m deep at the sampling location (samples taken at 0.5 m or 1 m). Samples from Mine 3 and Mine 1 TIs were collected off the end of docks ~4 m from shore. Mine 4 TI samples were collected from a boat ~20 m from shore.

Physicochemical and geochemical water samples from Mine 2 were collected and processed on-site within 1 h of collection, while water samples from Mine 1, Mine 3, and Mine 4 were shipped to the University of Toronto for processing, taking two to ten days potential shipping impacts addressed in Whaley-Martin et al. (2019). Depth samples from Mine 1 and Mine 2 were collected using a sterilized Van Dorn sampler as described in Whaley-Martin et al. (2019) and Mine 3 and Mine 4 samples were collected using a sterilized surface grab sampler. Water samples from Mine 1 and Mine 3 were collected in polyethylene liners (Uline S-1379) that were 70% ethanol sterilized and rinsed with target sample water three times prior to filling, using water directly from the Van Dorn sampler or surface grab sampler. Once 10 – 20 L were collected, liners were sealed without headspace, and placed in clean 20 L containers for shipping. Containers and liners remained unopened until arrival at the laboratory at the University of Toronto. Mine 2 water sample collection for microbial analysis followed the same protocol. Mine 4 water samples were collected into 70% ethanol sterilized and thrice rinsed (with target sample water) polyethylene containers before being submerged and filled directly leaving no headspace.

Each TI water sample was characterized for sulfur geochemistry and microbial community composition. Geochemical characterization included analyses of total sulfur (Total S), sulfate (SO_4_^2-^), thiosulfate (S_2_O_3_^2-^), and sulfite (SO_3_^2-^) concentrations and determination of reactive sulfur (S_React_; all S atoms capable of oxidation; determined by [Total S] – [SO_4_^2-^]; Whaley-Martin et al., 2020). Microbial community structure and function assessment included 16S rRNA and metagenomic analyses. Field measured TI water physicochemical parameters including temperature, pH, dissolved oxygen (DO; concentration and % saturation) and conductivity or salinity data were collected using a YSI 600 XLM (Mine 2), ProDSS water quality meter (Mine 1 and Mine 3), or a ThermoScientific Orion Star A329 Multiprobe (Mine 4).

Samples collected for 16S rRNA and metagenomic analyses were vacuum filtered (~2 to ~5 L) in triplicate through 0.1 μm and 0.2 μm filter units (Thermo Scientific™ Nalgene™ Rapid-Flow™ Sterile Disposable Filter Units with aPES Membrane) until clogged. Filters were immediately excised in a sterile Biological Safety Cabinet and kept frozen at -80°C until future extraction.

### 2.3 Geochemical analyses

Total sulfur samples were collected in triplicate by filtering 40 mL (per replicate) through a 0.45 μm filter (Pall Acrodisc^®^ 25 mm 0.45 μm Supor^®^ membrane filters) with polypropylene syringes into 50 mL Falcon™ tubes pre-spiked with 80 μL of HNO_3_ (Optima grade, Fisher Chemical). Unfiltered (UF) and 0.2 μm filtered total S samples were also collected, however, one-way ANOVA analysis revealed no statistically significant difference between the three filter fractions (*p* = 0.998) from these TI waters, therefore the 0.45 μm filter fraction data were used as these values represented the most complete dataset. These samples were stored at 4°C until they were shipped to the Commonwealth Scientific and Industrial Research Organization (CSIRO) for analyses using an inductively coupled plasma atomic emission spectroscopy (ICP-AES) on a Varian730 ES (Mulgrave, Australia). The limit of detection (LOD) for sulfur was 0.03 mM and concentrations were calculated by measuring intensity at the 181.972 nm sulfur emission line. Fast Automated Curve-fitting Technique (FACT) was used to correct for background and inter-element interferences.

100 μL aliquots of sample waters were preserved in triplicate for S_2_O_3_^2-^ and SO_3_^2-^ analysis using scaled derivatization methods described in Rethmeier et al. (1997). Samples were frozen until analysis. [S_2_O_3_^2-^] and [SO_3_^2-^] were quantified using a Shimadzu LC-20 AD prominence liquid chromatography (LC) system coupled with a fluorescence UV/VIS detector. An Alltima™ HP C18 reversed phase column (150 mm × 4.6 mm × 5 μm, Grace™) was used at 35°C using an isocratic mobile phase comprising 35% HPLC grade methanol and 65% of 0.25% acetic acid v/v (filtered, pH adjusted to 3.5 using NaOH) at 1 mL/min. The total run time was 12 min and the SO_3_^2-^ peak eluted at ~3.1 min and S_2_O_3_^2-^ eluted at ~3.4 min. The excitation wavelength used was 380 nm and the emission wavelength was 478 nm. Calibration curves were prepared using Na_2_SO_3_ (Sigma Aldrich, ≥ 98% purity) and Na_2_S_2_O_3_ (Sigma Aldrich, 99% purity). Limits of quantification for both S_2_O_3_^2-^ and SO_3_^2-^ were 0.01 mM.

Prior to 2019, SO_4_^2-^ samples were collected into clean sample bottles that were thrice rinsed (with target sample water) and filled leaving no headspace. Samples were stored at 4°C until analyzed. Aqueous dissolved SO_4_^2-^ was quantified by spectrophotometry using a HACH DR2800 (HACH Company, Loveland, CO, United States) using USEPA SulfaVer 4 Method 8051. SO_4_^2-^ samples collected in 2019 were 0.2 μm filtered (Pall Acrodisc^®^ 25 mm 0.2 μm Supor^®^ membrane filters) in triplicate using polypropylene syringes into 1.7 mL polypropylene vials and stored at 4°C until analyzed. Waters were analyzed for anions following USEPA Method 300.0/300.1 (only SO_4_^2-^ is reported here) on a Dionex™ ICS-6000 Capillary HPIC™ (high pressure ion chromatography; Thermo Scientific™, Part Number: ICS6000-003) System with a Dionex™ ICS-6000 CD Conductivity Detector (Thermo Scientific™, Part Number: 079829) and a Dionex IonPac™ AS18-Fast, 4 × 150 mm AS18 anion-exchange column (Thermo Scientific™, Part Number: 072062) and a Dionex IonPac™ AG18-Fast, 4 × 30 mm guard column (Thermo Scientific™, Part Number: 075762). A Dionex™ ICS-6000 EG Eluent Generator was used to produce the isocratic mobile phase of 23 mM KOH. Sample injection volume was 10 μL with sample aliquots diluted using 18.2 MΩ·cm deionized water when required. Matrix spikes and check standards were run every 20 samples and were also expected to fall withing ±15% of the expected value. The limit of detection for these samples was determined to be 0.008 mM dissolved SO_4_^2-^ with an instrument error of ±0.1 mM (Whaley-Martin et al., 2020; Yan et al., 2022).

Previous comparisons of the HACH spectrophotometry and IC methodology were completed on these waters by Whaley-Martin et al. (2020) identifying that dissolved [SO_4_^2-^] obtained by HACH spectroscopy and IC methods were consistent between methods for measured dissolved [SO_4_^2-^] between 0 and 250 mg/L.

### 2.4 16S rRNA sampling and statistical analyses

Genomic DNA was extracted from filters using Qiagen DNEasy PowerWater DNA Isolation Kits and the sample extracts were submitted to the McMaster University Genome Facility (Hamilton, Ontario, Canada) for subsequent analysis. Genomic DNA was quantified using quantitative PCR (polymerase chain reaction). Aliquots of purified DNA were used to amplify the V4 region of the 16S rRNA gene following the methods from Bartram et al. (2011) using Illumina primers and standards protocols from the Earth Microbiome Project (Caporaso et al., 2011, 2012). Briefly, the primers amplified the 515f (5′-GTGYCAGCMGCCGCGGTAA-3′) and 806r (5′-GGACTACNVGGGTWTCTAAT-3′) V4-variable regions of bacterial and archaeal 16S rRNA gene. 50 ng of DNA template was used for PCR and the PCR mix contained 1 U of recombinant Taq DNA Polymerase (Invitrogen™), 1x buffer, 1.5 mM MgCl_2_, 0.4 mg/mL bovine serum albumin (BSA), 0.2 mM deoxynucleotide triphosphate (dNTPs) and 5 pM of each primer. In accordance with Bates et al. (2010), the PCR reaction was as follows: initial denaturing at 98°C for 5 min, 35 cycles of denaturing at 98°C for 30 s, annealing at 50°C for 30 s and an extension at 72°C for 30 s with a final extension at 72°C for 10 min. The PCR products were confirmed using electrophoresis and sent for sequencing. A SequalPrep normalization kit (ThemoFisher #A1051001) was used to normalize all amplicons to 1.25 ng/L and then sequenced using the Illumina Mi-Seq.

DADA2 (v. 1.6.0) was used to check data for bimeras; 3 – 5% of the reads were determined to be bimeras and excluded from the dataset. Sequences which have undergone DADA2 denoising are referred to as amplicon sequence variants (ASVs). *Cutadapt* was used to filter and trim raw sequences using a minimum read length of 100 bp and used a minimum quality score of 30 (Martin, 2011). ASV tables were then merged and combined for each Illumina run and the SILVA database (v. 138.1) was used for taxonomic assignment using RStudio v. 1.4.1. While 16S rRNA calculated relative abundance is a widely employed method for assessing microbial community composition (e.g., Seth et al., 2024; Yu et al., 2024) due to its decreased cost, speed, and scalability (Durazzi et al., 2021), it is important to acknowledge potential sources of error inherent in this approach. These potential sources of errors include variability in 16S gene copy number and amplification/sequencing bias and error (Suzuki and Giovannoni, 1996; Hong et al., 2009; Kembel et al., 2012). However, their combination with other independent lines of evidence, i.e., metagenome and geochemical data, can assist in robust understanding of microbial community composition, function, and environmental preferences (Louca et al., 2018; Campa et al., 2022; Whaley-Martin et al., 2023).

Shannon Diversity index and sample richness values were calculated in RStudio v. 1.4.1 based on the number of unique amplicon sequence variant (ASVs) and sequence abundances. Data visualization and linear regression (Pearson’s correlations and associated ANOVA tests) were performed in OriginPro v. 2022. Additional ANOVA and post-doc Tukey pairwise statistical analyses were performed in RStudio v. 1.4.1.

### 2.5 Metagenomic sequencing, reads processing, and assembly

The genomic DNA was extracted from the sample filters individually and used for subsequent metagenomic sequencing. The DNA extracts were dried and resuspended in 25 μL aliquots of water. The Illumina library preparation kits were used in the construction of sequencing libraries with an insert length of ~500 bp. The libraries were sequenced using the Illumina HiSeq-1500 platform with paired-end 150 bp sequencing kits by the Farncombe Metagenomics Facility at McMaster University (Hamilton, Ontario, Canada), as previously described in Whaley-Martin et al. (2023).

The raw sequencing reads were filtered to remove Illumina adapters, PhiX and other Illumina trace contaminants using BBTools (Bushnell et al., 2017) and low quality bases and reads were removed using Sickle (v. 1.33). All reads with both ends remained were used for subsequent *de novo* assembly via IDBA_UD (parameters: --mink 20, --maxk 140, --step 20, --pre_correction; Peng et al., 2012) or metaSPAdes (parameters: -k 21,33,55,77,99,127; Nurk et al., 2017). Sequencing coverage for each scaffold from a given sample was individually mapped using quality paired-end reads to the full assembly using Bowtie2 with default parameters (Langmead and Salzberg, 2012). Generated sam files were converted to bam format and sorted using samtools (Li et al., 2009). Then the coverage of each scaffold was calculated using the script of jgi_summarize_bam_contig_depths from MetaBAT (Kang et al., 2015). Using MetaBAT, all scaffolds with a minimum length of 2,500 bp were assigned to genome bins with both tetranucleotide frequency and sequencing coverage profiles from all samples considered. Both binned and unbinned scaffolds with a minimum length of 1,000 bp were uploaded to ggKbase^1^ for manual genome bin refinement, which was based on GC content, sequencing coverage, and taxonomic information of each scaffold as previously described in Chen et al. (2020) and was primarily based on patterns and distribution of GC content and sequencing coverage of contigs in each bin. Contamination contigs/scaffolds were removed from metagenome assembled genomes using ggKbase if the contig/scaffold had divergent sequencing coverage (those with <0.5x or > 2x coverage relative to most other contigs/scaffolds in the metagenome assembled genome), GC content, and/or divergent taxonomic assignment. Taxonomic assignment of metagenome assembled genomes was determined based on taxonomic classification of protein-coding genes within each contig or scaffold. This process involved identifying the best taxonomic matches of the protein-coding genes to references proteins and determining the last common ancestor where ≥50% of the matches align with a particular taxonomic group. Overall taxonomic assignment of a metagenome assembled genome was then derived from the cumulative taxonomic assignments of all its contigs/scaffolds.

#### 2.5.1 Gene prediction and annotation

Gene prediction and subsequent analyses were completed using assembled scaffolds with a minimum length of 1,000 bp (herein “1k_scaffolds) to assist in obtaining metagenome assembled genomes with minimal contamination and high completeness. The protein-coding genes were predicted using Prodigal v. 2.6.3 from 1k_scaffolds (parameters: -m -p meta; Hyatt et al., 2010). 16S rRNA genes were predicted from 1k_scaffolds based on an HMM database in accordance with methods presented by Brown et al. (2015). The tRNAs on all 1k_scaffolds were predicted using tRNAscanSE (v. 2.0.3; Chan and Lowe, 2019). For functional annotation, the predicted protein-coding genes were searched against the Kyoto Encyclopedia of Genes and Genomes databases (KEGG; Kanehisa et al., 2017), UniRef100 (Suzek et al., 2007), and UniProt (Apweiler et al., 2004) via Usearch (v.10.0.240_i86linux64; Edgar and Bateman, 2010). HMM databases (Anantharaman et al., 2016) and KOfam HMM database (Kanehisa and Sato, 2020) were used to search and identify the predicted protein-coding genes for specific metabolic potentials of interest. DiSCo (Neukirchen and Sousa, 2021) was used to distinguish *dsrC* and its homologs. BLASTp was used to search predicted proteins against the shdr protein sequences encoded by *Acidithiobacillus caldus* SM-1, *Thioalkalivibrio* sp. K90mix, and *Hyphomicrobium denitrificans* (Koch and Dahl, 2018) followed by manual confirmation. Screening for *hdrAACB* genes was completed by checking the annotations from KEGG, UniRef100, and UniProt using the key word “hdr” and the acquired list of genes was manually confirmed. The *aprM* and *aprBA* genes were identified by comparing all protein-coding genes (from corresponding metagenomes) against *aprM*/*aprBA* identified in *Thiobacillus denitrificans* (WP_011312796.1) using BLASTp with an e-value threshold of 1e-10 and hits were manually verified. The *tetH* gene was identified using a BLASTp search as previously described in Watanabe et al. (2019) followed by manual verification. Failure to detect genes which require annotated reference genomes (s*hdr, hdrAACB, aprM, aprBA*, and *tetH*) for detection, rather than KO values (or other database values), may result in under-reporting of those genes until more annotated reference genomes are reported in the literature.

## 3 Results and discussion

### 3.1 SOB community 16S rRNA composition and metagenomic inferred function

Microbial community composition was determined using 16S rRNA relative abundances for each TI sample (*n* = 13 from Mine 1, *n* = 15 from Mine 2, *n* = 6 from Mine 3, and *n* = 8 from Mine 4). Nine major genera of SOB were identified in these 42 TI samples including: *Halothiobacillus* spp. (12.7 ± 20.5%), *Sediminibacterium* spp. (4.8 ± 9.1%), *Thiobacillus* spp. (3.9 ± 7.8%), *Sulfuricurvum* spp. (3.7 ± 11.5%), *Thiovirga* spp. (1.5 ± 3.2%), *Sulfuritalea* spp. (0.7 ± 2.9%), *Sulfurimonas* spp. (0.4 ± 1.3%), *Sulfuriferula* spp. (0.4 ± 0.7%), and *Thiomonas* spp. (0.1 ± 0.2%). Each genus occurred at >1% abundance in at least one sample (Table 2). The presence and relative abundance of these nine SOB genera differed among mines and over time.

**Table 2:** Average (± standard deviation), minimum and maximum 16S rRNA percent relative abundances across sulfur oxidizing bacteria genera data for Mine 1, Mine 2, Mine 3, and Mine 4 tailings impoundment waters (2016 – 2019).

	Mine 1	Mine 2	Mine 3	Mine 4
*Thiomonas* spp. Abundance (%)	0.0 – 0.7 (avg. = 0.1 ± 0.2)	0.0 – 1.2 (avg. = 0.1 ± 0.3)	0.0 – 0.01 (avg. = 0.002 ± 0.01)	0
*Halothiobacillus* spp. Abundance (%)	0.0 – 24 (avg. = 2.3 ± 6.3)	0.2 – 64 (avg. = 34 ± 21)	0.01 – 0.1 (avg. = 0.05 ± 0.04)	0.0 – 0.1 (avg. = 0.02 ± 0.02)
*Thiovirga* spp. Abundance (%)	0.0 – 6.1 (avg. = 1.6 ± 2.2)	0.0 – 17 (avg. = 1.9 ± 4.1)	0.0 – 10 (avg. = 1.8 ± 3.8)	0.0 – 1.1 (avg. = 0.2 ± 0.4)
*Sulfuricurvum* spp. abundance (%)	0.0 – 8.2 (avg. = 1.4 ± 2.9)	0.01 – 55 (avg. = 9.2 ± 18)	0.002 – 1.0 (avg. = 0.2 ± 0.4)	0.0 – 0.1 (avg. = 0.02 ± 0.04)
*Sulfurimonas* spp. abundance (%)	0.0 – 8.0 (avg. = 1.2 ± 2.2)	0.0 – 0.2 (avg. = 0.1 ± 0.1)	0.0 – 0.1 (avg. = 0.01 ± 0.02)	0.0 – 0.02 (avg. = 0.003 ± 0.01)
*Thiobacillus* spp. abundance (%)	0.0 – 39 (avg. = 7.0 ± 12)	0.0 – 14 (avg. = 1.1 ± 3.4)	0.1 – 12 (avg. = 5.1 ± 4.8)	0.2 – 12 (avg. = 3.0 ± 3.6)
*Sediminibacterium* spp. abundance (%)	0.0 – 27 (avg. = 5.3 ± 9.0)	0.0 – 48 (avg. = 6.5 ± 12)	0.1 – 12 (avg. = 3.5 ± 3.9)	0.0 – 13 (avg. = 1.7 ± 4.3)
*Sulfuriferula* spp. abundance (%)	0.0 – 0.9 (avg. = 0.1 ± 0.2)	0.04 – 3.1 (avg. = 0.6 ± 0.8)	0.0 – 0.5 (avg. – 0.1 ± 0.2)	0.0 – 2.4 (avg. = 0.7 ± 0.9)
*Sulfuritalea* spp. abundance (%)	0.0 – 0.1 (avg. = 0.01 ± 0.02)	0	0.0 – 18 (avg. = 3.2 ± 6.6)	0.0 – 5.2 (avg. = 1.2 ± 2.0)
Relative abundance of SOB (%)	0.0 – 65 (avg. = 19 ± 23)	12 – 77 (avg. = 53 ± 19)	1.6 – 23 (avg. = 14 ± 7.5)	1.0 – 19 (avg. = 6.7 ± 6.4)

**Figure 1:**
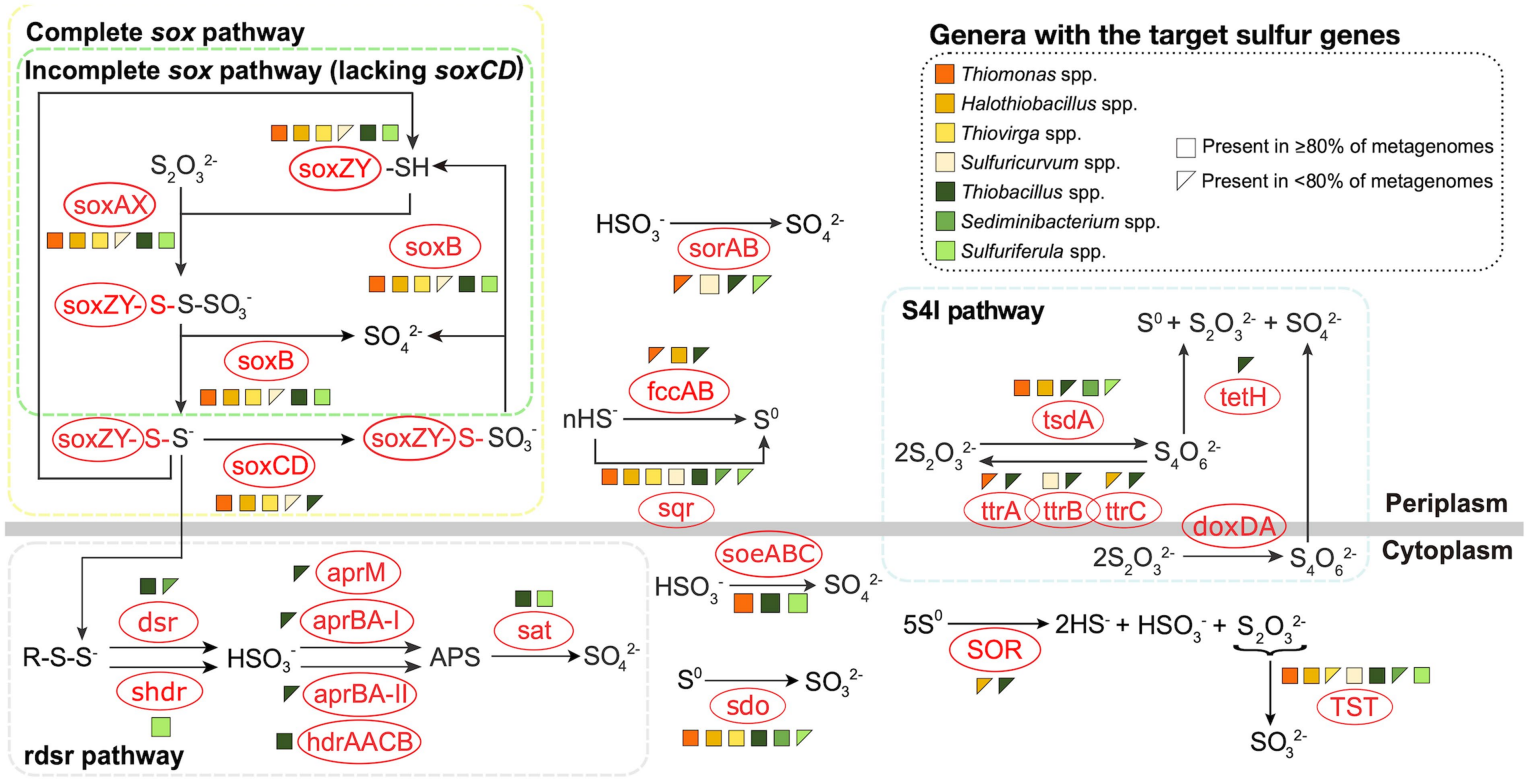
Metabolic potentials and pathways for sulfur oxidizing bacteria genera from the four TIs investigated here. Figure was adapted from Whaley-Martin et al. (2023) and based on Watanabe et al. (2019). Genomes were reconstructed from TI samples collected from the four target mines between 2016 and 2018. Due to low abundance of some organisms, variable quantities of genomes were used including 3 *Thiomonas*, 31 *Halothiobacillus*, 3 *Thiovirga*, 31 *Thiobacillus*, 30 *Sediminibacterium*, 4 *Sulfuricurvum*, and 14 *Sulfuriferula*.

To identify the potential sulfur metabolizing pathways encoded by these SOB genera, 116 metagenomes were constructed from 38 samples collected at these four mines between 2016 and 2018 (Supplementary Table 2). Genomes for *Sulfurimonas* and *Sulfuritalea* could not be reconstructed and thus interpretations for these two genera relied on literature reports. Three major pathways including *sox*, r*dsr*, and S_4_I were examined, as well as additional sulfur oxidation genes not specific to those three pathways (Figure 1), based on the foundational understanding outlined by Watanabe et al. (2019) and expanded by Whaley-Martin et al. (2023). Genes encoding the *sox* pathway, either complete (c*sox*; *soxXYZABCD*) or incomplete (i*sox*; *soxXYZAB* and lacking *soxCD*) were most common – occurring in eight of the nine genera. *Thiomonas*, *Halothiobacillus,* and *Thiovirga* genomes encoded the c*sox* pathway (resulting in generation of SO_4_^2-^; Figure 1) which is consistent with published reports for *Thiomonas* and *Halothiobacillus* (Veith et al., 2012; Lin et al., 2015). Outside of other published works from the mines included in this study, there are very limited data available in the literature for *Thiovirga* spp., Miettinen et al. (2021) identified possible *Thiovirga* spp. in zinc and copper ore processing facilities in Portugal that lacked only the *soxC* subunit, indicating there may be variability within the *Thiovirga* genus regarding the ability for complete oxidation to SO_4_^2-^ via the *sox* pathway, unlike the findings presented here (Figure 1). *Sulfurimonas* spp. have been reported to often possess the *soxCD* genes (i.e., c*sox*) (Lahme et al., 2020; Wang et al., 2021, 2023) however, this genus may lack *soxAB* genes instead, which would not allow for complete oxidation via the *sox* pathway (Lahme et al., 2020; Wang et al., 2021). At least one strain of *Sulfurimonas* (Strain NW10^T^) has been reported to possess genes for the c*sox* pathway but used the i*sox* pathway instead (Wang et al., 2021).

*Sediminibacterium* was the only detected SOB in this study that did not encode any *sox* genes (Figure 1). Two genera were identified as possessing the i*sox* pathway; *Sulfuriferula* and *Thiobacillus* (Figure 1). Watanabe et al. (2019) reported *Sulfuritalea hydrogenivorans* sk43H, which at present is the only published pure culture strain, as possessing *soxAX*, *soxYZ*, and *soxB* but lacking *soxCD,* thus indicating the presence of the i*sox* pathway. Three of the reconstructed *Sulfuricurvum* spp. genomes encoded c*sox,* while one encoded no *sox* genes (Figure 1), with the latter potentially due to the low quality of the genome. This finding of c*sox* encoding *Sulfuricurvum* from mining TI samples described here, and also reported in Whaley-Martin et al. (2023), diverges from the current literature which typically reports *Sulfuricurvum* spp. found in the natural environment (terrestrial aquifer, geothermal springs) as possessing the i*sox* pathway (lacking *soxCD*; Handley et al., 2014; Meziti et al., 2021).

A S_4_I pathway gene for the first reaction generating S_4_O_6_^2-^, was the second most abundant identified across the seven genera with reconstructed genomes (Figure 1). Two distinct catalysts, *tsdA* and *doxDA,* are responsible for the conversion of S_2_O_3_^2-^ to S_4_O_6_^2-^ (Nguyen et al., 2022). However, *doxDA* was absent in the reconstructed genomes, while *tsdA* was present in the genomes of five of the seven genera, including >80% of *Thiomonas*, *Halothiobacillus*, and *Sediminibacterium* genomes and < 80% of *Thiobacillus* and *Sulfuriferula* genomes (Figure 1). Generation of tetrathionate from *tsdA* activity can be subsequently disproportionated via *tetH* to form S^0^, S_2_O_3_^2-^, and SO_4_^2-^, which can then potentially feed both the incomplete/complete *sox* pathway and/or S^0^ storage pathways (including *sdo*, *SOR,* and r*dsr*; Friedrich et al., 2005; Frigaard and Dahl, 2008; Watanabe et al., 2019). Though *tsdA* was found in five genomes, *tetH,* which would be required for the completion of the S_4_I pathway, was only found in five of the thirty-one *Thiobacillus* genomes (<80%; Figure 1). This may indicate the potential for genera with only *tsdA* (such as *Thiomonas*, *Halothiobacillus*, *Sediminibacterium* and *Sulfuriferula*) to couple with *tetH* encoding *Thiobacillus* to complete the S_4_I pathway. Limited *tetH* detection may also reflect current limitations in *tetH* reference material. *TtrABC* can catalyze the reduction of S_4_O_6_^2-^ to produce S_2_O_3_^2-^ though its components (*ttrA, ttrB*, and *ttrC*) were only variably present across *Thiobacillus* genomes (Figure 1). *Thiomonas* spp., *Sulfuricurvum* spp., and *Halothiobacillus* spp. were found to possess *ttrA, ttrB*, and *ttrC* respectively, but there is no available research indicating these subunits are active in sulfur reduction individually. Watanabe et al. (2019) reported that *doxDA*, *tsdA*, and *tetH* genes were absent in *Sulfuritalea hydrogenivorans* sk43H indicating this *Sulfuritalea* species did not encode the S_4_I pathway. Currently, there is a lack of specific data regarding the presence or activity of the S_4_I pathway (and associated genes *tsdA*, *doxDA*, and *tetH*) within *Sulfurimonas*. Consequently, it remains uncertain in results here and in available literature, whether *Sulfurimonas* possess the genomic capacity to support the S_4_I pathway.

The genes for the complete *rdsr* pathway were detected in 29 of the 31 *Thiobacillus* genomes (Figure 1). *Sulfuriferula* possessed the potential to express *shdr* (SO_3_^2-^ production) and *sat* (SO_4_^2-^ production through APS) but lacked the intermediate *aprM/aprBA-*I or *aprBA-II/hdrAACB* genes that mediate the reaction produce APS (Figure 1), aligning with results presented in Watanabe et al. (2019) for *Sulfuriferula* sp. AH1 and *Sulfuriferula thiophila* mst6. These missing steps could be compensated for by the cytoplasmically oriented *soeABC* that was present in all *Sulfuriferula* genomes, which generates SO_4_^2-^ directly from SO_3_^2-^ (Koch and Dahl, 2018; Figure 1). Alternatively, SO_3_^2-^ can be transported to the periplasm via *TauE*-like transporter where it can spontaneously react with hydrogen sulfide if present (Koch and Dahl, 2018). The lack of *rdsr* and *shdr* pathway genes in the SOB identified in this study, may reflect the relatively high dissolved oxygen concentrations across these water samples (averaging between 36.2 – 82.6 % saturation across the four mines; Table 1) which do not favour establishment of anaerobic and/or microaerophilic SOB which typically harbour the *rdsr* pathway (Klatt and Polerecky, 2015). Isolated genomes of *Thiobacillus* contained *aprM*, *aprBA-I*, *aprBA-II*, and/or *hdrAACB,* which would allow them to further oxidize SO_3_^2-^ to APS (Figure 1). Watanabe et al. (2019) reported the presence of partial (lacking *aprM* and *aprBA-I*) *rdsr* pathway genes (including *dsrAB*, *dsrEFH*, *dsrC, dsrMKJOP,* and *sat*) in the model species *Sulfuritalea hydrogenivorans sk43H*. Furthermore, evidence reported by Purcell et al. (2014) indicates *Sulfuritalea hydrogenivorans sk43H* can carry out the *rdsr* pathway within Antarctic lake sediments. *Sulfuritalea hydrogenivorans sk43H* also hosts a partial s*hdr* pathway including *aprBA-II* and *hdrAACB* (Watanabe et al., 2019). Second only to the *Thiobacillus* genomes presented here, *Sulfuritalea hydrogenivorans sk43H* hosts the most diverse set of r*dsr* pathway genes of the nine SOB genera identified. While there are several published *Sulfurimonas* genomes, detailed insights into r*dsr* pathway occurrence remains limited. This may be due to the absence of the *rdsr* pathway within the *Sulfurimonas* genus as Wang et al. (2023) reports *Sulfurimonas* sp. ST-27 lacks *dsrAB*, *dsrE*, *dsrCD*, *dsrMKJOP,* and *aprBA,* but does contain *sat*. The *rdsr* and *shdr* pathways may play an important role in bacteria harbouring the i*sox* pathway by facilitating the conversion of sulfane sulfur, produced as an intermediate, into SO_4_^2-^ (Xin et al., 2023). SOB that encode the i*sox* pathway without the r*dsr* or s*hdr* pathways, may produce and accumulate sulfane sulfur as a by-product of S_2_O_3_^2-^ oxidation, though the mechanism for reducing oxidative stress from the accumulation of sulfane sulfur is not well delineated and may result in the accumulation of volatile H_2_S (Xin et al., 2023). Additionally, sulfane sulfur has been found to exhibit toxicity in both bacteria and fungi, including when produced through the i*sox* pathway in *Cupriavidus pinatubonensis* (Sato et al., 2011; Xin et al., 2023). Of the four genera in this study which contain the i*sox* pathway, only *Sulfuritalea* and *Thiobacillus* possess the ability to detoxify sulfane sulfur using either the r*dsr* or s*hdr* pathway.

Additional cytoplasmic sulfur oxidative genes investigated include *TST* (S_2_O_3_^2-^ to SO_3_^2-^) and *soeABC* (SO_3_^2-^ to SO_4_^2-^; Figure 1). *TST* was broadly present across the SOB occurring in >80% of the *Thiomonas* spp., *Halothiobacillus* spp., *Thiobacillus* spp., *Sulfuricurvum* spp., and *Sulfuriferula* spp. metagenomes and in <80% of the *Thiovirga* spp. and *Sediminibacterium* spp. metagenomes (Figure 1). To date, no information is available regarding the presence or absence of *TST* in *Sulfurimonas* or *Sulfuritalea* genomes. Wang et al. (2019) suggests *TST* may use S_2_O_3_^2-^ produced by *SOR* to further oxidize it to sulfur and SO_3_^2-^. *SoeABC* transforms SO_3_^2-^ to SO_4_^2-^ in the cytoplasm and may even contribute to SO_3_^2-^ oxidation as part of the r*dsr* pathway (Dahl et al., 2013; Whaley-Martin et al., 2023). Greater than 80% of the *Thiomonas, Thiobacillus* and *Sulfuriferula* genomes contained *soeABC* (Figure 1). *SoeABC* has also been identified in *Sulfuritalea hydrogenivorans* sk43H (Watanabe et al., 2014) but no information on *soeABC*’s occurrence in *Sulfurimonas* is currently available. Additional periplasmic sulfur oxidation genes identified in this study include *sqr*, *fccAB,* and *sorAB* (Figure 1). Both *sqr* and *fccAB* are implicated in catalysis of hydrogen sulfide to S^0^ which can form S^0^ globules and be further oxidized to SO_3_^2-^ via the r*dsr* pathway (Nosalova et al., 2023). *Sqr* was widely present appearing in >80% of the genomes identified in all genera apart from *Sediminibacterium* and *Sulfuriferula* metagenomes where they were present in <80% of the metagenomes (Figure 1). *Sulfuritalea hydrogenivorans* sk43H and various *Sulfurimonas* species were found to host the s*qr* gene (Watanabe et al., 2019; Wang et al., 2021, 2023). Among the reconstructed genomes, >80% of the *Halothiobacillus* genomes harbored the *fccAB* gene cluster while the *fccAB* cluster was identified in <80% of the *Thiomonas* and *Thiobacillus* genomes (Figure 1). *Sulfuritalea hydrogenivorans* sk43H has been reported to contain *fccAB* (Watanabe et al., 2019) and currently no *Sulfurimonas* species have been reported to contain *fccAB*. *SorAB*, which catalyzes the conversion of SO_3_^2-^ to SO_4_^2-^ in the periplasm, was found in >80% of the *Sulfuricurvum* genomes and < 80% of the *Thiomonas, Thiobacillus*, and *Sulfuriferula* genomes (Figure 1). *SorAB* has also been reported in *Sulfuritalea hydrogenivorans* sk43H (Watanabe et al., 2019) as well as eight of the eleven *Sulfurimonas* species examined by Wang et al. (2021). The four most abundant genera in these four TI collectively possess the capacity for sulfur oxidation via all three universal pathways, c*sox* (*Halothiobacillus*, *Sulfuricurvum*), i*sox* + *rdsr* (*Thiobacillus*), and S_4_I (*Halothiobacillus, Sediminibacterium*, *Thiobacillus*) suggesting adaptation of these TI SOB communities to occupy all potential sulfur oxidizing niches that occur in these highly physicochemically and geochemically dynamic TI systems.

#### 3.1.1 SOB functional classification

Under acid mine drainage conditions (acidic, metal rich), Kuang et al. (2016) identified metabolic function as a better predictor of microbial community structure and function than taxonomy. Similarly, here, patterns in metagenomic data were used to classify these SOB genera into c*sox* dominant and non-c*sox* dominant SOB genera groupings. C*sox* dominant SOB genera identified here included *Halothiobacillus* spp., *Thiovirga* spp., *Thiomonas* spp., and *Sulfuricurvum* spp. based on the presence and high abundance of the c*sox* pathway (Figure 1). Non-c*sox* dominant SOB genera were characterized by i*sox* gene pathway presence and increased abundance of genes associated with alternative pathways such as r*dsr* (*dsrABCEFH*, *aprAB*, *sat*; Whaley-Martin et al., 2023) and/or S_4_I (*tsdA, tetH*; Ghosh and Dam, 2009; Wang et al., 2019; Whaley-Martin et al., 2023) (Figure 1). *Thiobacillus* spp., *Sediminibacterium* spp., *Sulfuriferula* spp., *Sulfuritalea* spp., and *Sulfurimonas* spp. were categorized as non-c*sox* dominant SOB genera (Figure 1). Importantly, reactions catalyzed by the c*sox* pathway favour the complete oxidation of SOI to SO_4_^2-^, resulting in increased acidity and SO_4_^2-^ production, while more energy efficient pathways (e.g., r*dsr*; Klatt and Polerecky, 2015), favoured by non-c*sox* dominant SOB commonly generate free SOI and in some cases may consume H^+^ (Dam et al., 2007; Klatt and Polerecky, 2015; Hutt et al., 2017). Therefore, presence or absence of c*sox* vs. non-c*sox* dominant pathways may reflect, and in turn, result in physicochemically and geochemically distinct waters. Mine waters with non-c*sox* dominant SOB (i.e., *Thiobacillus*, which can generate free SOI) may be at greater risk of offsite acidification due to the ability of many SOI to pass through current treatment to receiving environments where their subsequent oxidation could release acidity.

#### 3.1.2 Spatial and temporal trends in SOB community composition and function

**Figure 2:**
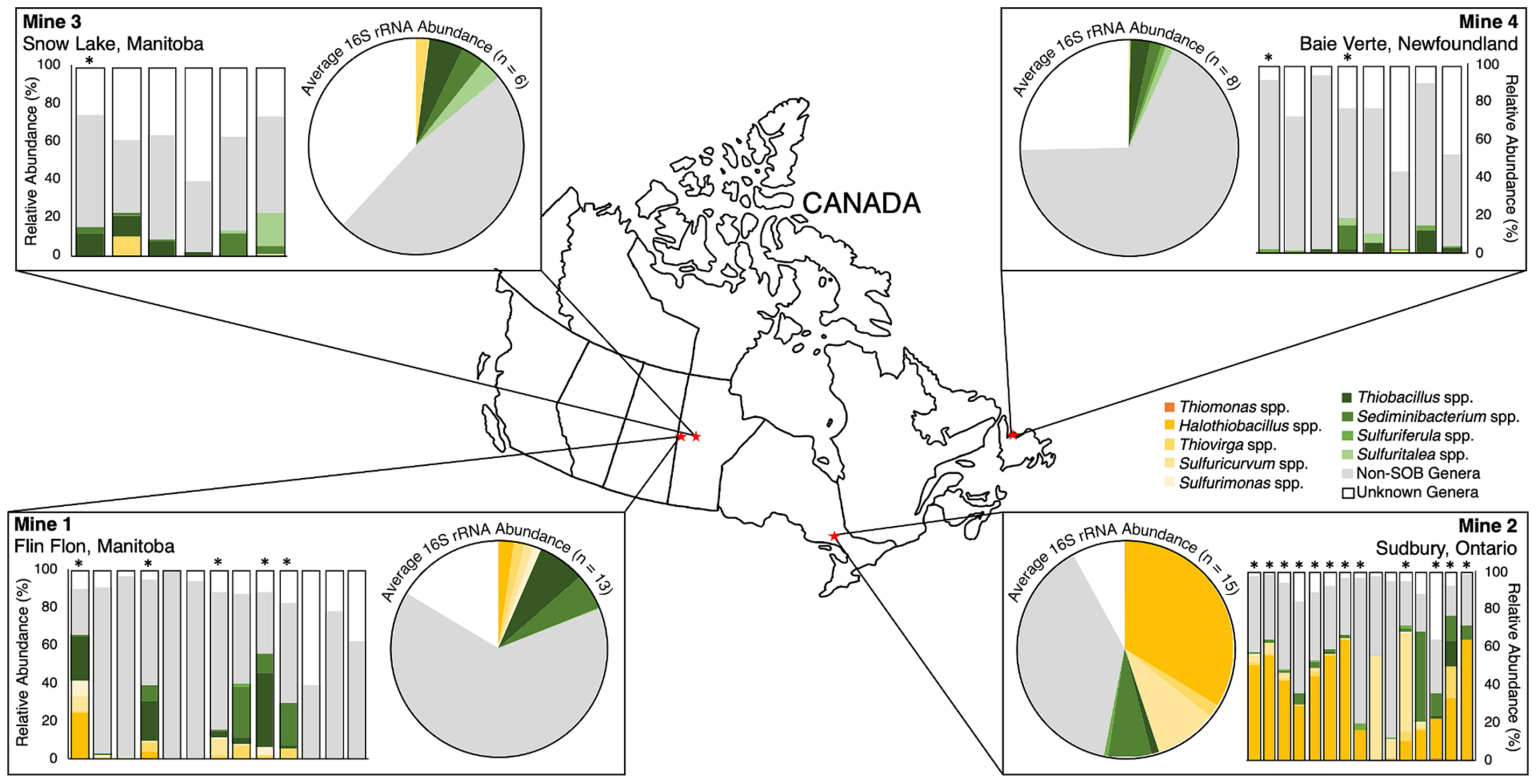
16S rRNA relative abundance (%) of top nine sulfur oxidizing bacteria genera from 2016 – 2019, non-SOB genera and unknown genera for each of the four mines mapped to their sample locations across Canada. Individual samples are shown as bar graphs and mine average abundances are shown as pie graphs. “Non-SOB genera” are all other identified sequences and “unknown genera” are identified sequences not matched to genera in the Silva Database v138.1. Asterisks (*) denote samples with metagenome data included in this study.

The abundance of the nine identified SOB genera differed spatially and temporally for individual mines as well as across the four mines (Figure 2). The two oldest TIs, Mine 2 and Mine 1, had the highest SOB abundances (Figure 2). Mine 2 TI had the largest total abundance of SOB genera across all samples (54 ± 19%) and was the only mine TI where c*sox* dominant SOB genera (predominantly *Halothiobacillus* spp.) had a higher average abundance than non-c*sox* dominant SOB genera (predominantly *Sediminibacterium* spp.; Figure 2, Table 2). With more than 50% of the community on average consisting of SOB, Mine 2 TI had the lowest non-SOB genera abundance (38 ± 17%) and lowest unknown genera abundance (12 ± 9.4%; Figure 2). Based on evidence from this study and a previous study (Whaley-Martin et al., 2023), the Mine 2 community had the potential to express the c*sox* pathway (via *Halothiobacillus* spp., *Thiovirga* spp., *Thiomonas* spp. or *Sulfuricurvum* spp.), i*sox* pathway (via *Thiobacillus* spp. or *Sulfuriferula* spp.), r*dsr* pathway (via *Thiobacillus* spp.), and/or the S_4_I pathway (via *Thiobacillus* spp.). The average c*sox* dominant SOB abundance was significantly higher in Mine 2 (*p* < 0.001) compared to the other three mines, while the abundance of non-c*sox* dominant SOB were not significantly different across the four mines. Averaging a total SOB abundance 2.7 times lower than Mine 2, Mine 1 TI had the second highest total SOB abundance (20 ± 24%) (Figure 2, Table 2). SOB genera present at Mine 1 could express the c*sox* pathway (via *Halothiobacillus* spp., *Thiovirga* spp., or *Sulfuricurvum* spp.), i*sox* pathway (via *Thiobacillus* spp. or *Sulfurimonas* spp.), r*dsr* pathway (via *Thiobacillus* spp.), and/or the S_4_I pathway (via *Thiobacillus* spp.).

Mine 3’s TI waters averaged 14 ± 7.5% total SOB abundance (Table 2), which was approximately 4 times lower than Mine 2 and 1.4 times lower than Mine 1. Mine 3 TI did, however, have the highest abundance of unknown genera (38 ± 12%) and the second highest abundance of identified non-SOB genera (48 ± 8.2%; Figure 2). The majority of TI SOB present in Mine 3 TI waters were identified as non-c*sox* dominant SOB and averaged 12 ± 6.2% primarily consisting of *Thiobacillus* spp., *Sediminibacterium* spp. and *Sulfuritalea* spp. (Figure 2, Table 2). The average abundance of c*sox* dominant SOB genera at Mine 3 was 2.0 ± 3.7% and was primarily *Thiovirga* spp. (Figure 2, Table 2). 16S rRNA abundance, metagenome data and present literature indicate that SOB genera from Mine 3 could express the c*sox* pathway (via *Thiovirga* spp.) and/or the i*sox* (via *Thiobacillus* spp. or *Sulfuritalea* spp.), r*dsr* (via *Thiobacillus* spp.), and S_4_I pathway (via *Thiobacillus* spp.).

The lowest total SOB abundance was found in the Mine 4 TI samples, with an average abundance of 7.1 ± 6.3 and > 97% of the SOB present represented by non-c*sox* dominant SOB genera (Table 2). Non-c*sox* dominant SOB genera averaged 6.5 ± 6.6% of the overall community abundance, while c*sox* dominant SOB abundance was the lowest of the four mines averaging 0.3 ± 0.4%, with only *Thiovirga* spp. ever reaching above 1% of the overall community abundance at Mine 4 (Table 2). Mine 4 had the second highest contribution of unknown genera representing 27 ± 17% of the microbial community (Figure 2). Mine 4 had the most limited S oxidation potential with no major SOB c*sox* abundance (Table 2). *Thiobacillus* spp. present at Mine 4, contained *tsdA* enabling part 1 of the S_4_I pathway (S_4_O_6_^2-^ formation), but did not contain *tetH* associated with the 2^nd^ part of the S_4_I pathway (S_4_O_6_^2-^ disproportionation) and did contain genes for the i*sox* and r*dsr* pathway.

### 3.2 TI wastewater sulfur geochemistry

**Figure 3:**
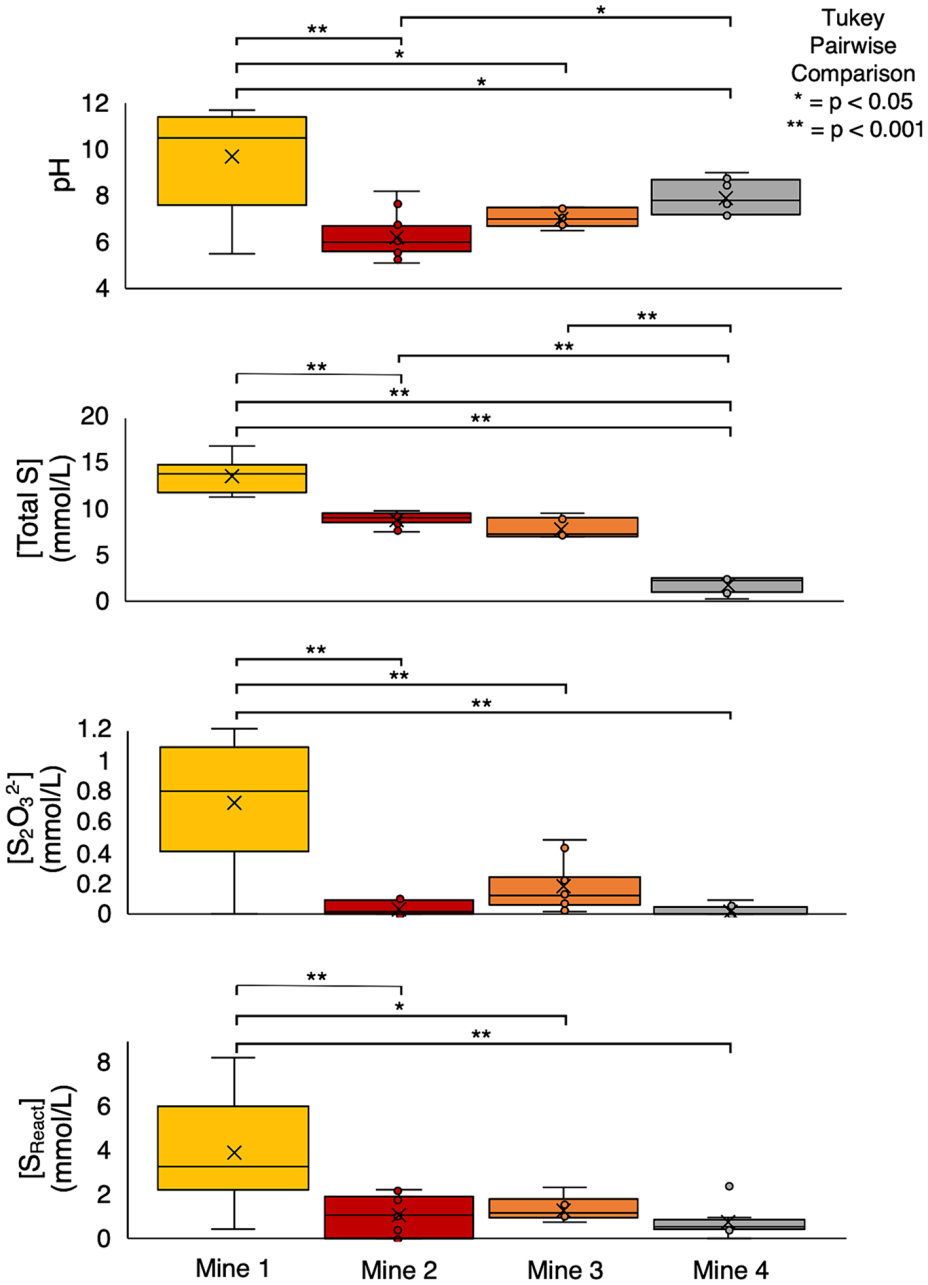
Box and whisker plots and statistical analysis (ANOVA and post-hoc tukey pairwise statistical comparison) of cross mine pH, total S (mmol/L), S_2_O_3_^2-^ (mmol/L), and S_React_ (mmol/L). Box limits represent the first and third quartile of each dataset, with a black line indicating the median value and an “x” denoting the mean.

Mean [total S_0.45μm_] and [SO_4_^2-^] across the four mines display a pattern of increasing concentration with age, though this relationship is not proportional. Notably, Mine 1 and Mine 2 are most similar in age and Mine 2 and Mine 3 are most similar in concentrations (Table 1, Figure 3). However, [total S_0.45μm_] exhibited substantial variability across the four mines – ranging from 0.3 mM (Mine 4) to 16.8 mM (Mine 1) (Table 1). ANOVA and post-hoc Tukey pairwise comparison tests revealed a significantly higher average total S_0.45μm_ concentration at Mine 1 (14 ± 1.6 mM) compared to Mine 2 (8.9 ± 0.7), Mine 3 (7.9 ± 1.0), and Mine 4 (1.9 ± 0.8 mM) (Table 1; *p* < 0.001). [SO_4_^2-^] displayed a similar trend where Mine 1 exhibited significantly higher concentrations than Mine 2 (*p* < 0.05), Mine 3 (*p* < 0.001), and Mine 4 (*p* < 0.001) (Table 1, Figure 3). Elevated sulfur concentrations in Mine 1 and Mine 2 TIs can be attributed to their extensive use since the 1920s. Mine 3, while younger than Mine 1 and Mine 2, contains a comparatively higher [total S_0.45μm_], reflecting tailings additions from multiple mining operations and a shallow water cover depth. Mine 4, the smallest and youngest TI, has accumulated the lowest volume of tailings, resulting in the lowest [total S_0.45μm_].

Though not often reported, [total S] from various environments varies widely from ~10 μM to 800 μM observed in freshwater lakes (Vincent et al., 2021) to ~28 mM in sea water (Canfield and Farquhar, 2009; Vincent et al., 2021) with SO_4_^2-^ typically being the largest contributor to the overall sulfur balance (Canfield and Farquhar, 2009). Reported [SO_4_^2-^] from other mining environments studies range from 3.6 mM to >250 mM (Foucher et al., 2001; Silva et al., 2012; Kinnunen et al., 2018; Camacho et al., 2020b). [Total S] (0.3 – 16.8 mM; Table 1) in this study are typically higher than the reported freshwater <1 μM value, while on the lower end of reported values for mining contexts (Foucher et al., 2001; Kinnunen et al., 2018).

Reactive sulfur concentrations ([S_React_] calculated as [Total S] – [SO_4_^2-^]; Whaley-Martin et al., 2020), representing all sulfur atoms capable of oxidation, were determined for each TI sample. ANOVA and post-hoc Tukey pairwise comparison tests revealed that Mine 1 had a significantly higher average S_React_ compared to Mine 2 (*p* < 0.001), Mine 3 (*p* < 0.05) and Mine 4 (*p* < 0.001) (Figure 3). Considering the potential 10-fold variation in both [total S_0.45μm_] and [S_React_] between samples, %S_React_ ([S_React_]/[Total S_0.45μm_] x 100) provides a useful metric to reflect the proportion of S_React_ within each system’s individual total S pool. %S_React_ ranged from 0 to 92.3% across the four mines, with the highest average %S_React_ occurring at Mine 1 (28 ± 16.2%) followed by Mine 4 (27 ± 13%), Mine 3 (17 ± 4.4%), and Mine 2 (12 ± 9.5%). S_React_ may include various sulfur species such as S_2_O_3_^2-^ and SO_3_^2-^ (quantified in this study), as well as additional unresolved sulfur species including but not limited to S_4_O_6_^2-^, S_2_O_4_^2-^, and S^0^ which were not quantified here but could potentially contribute to or support microbial sulfur cycling (Bak and Pfennig, 1987; Kelly et al., 1997). [SO_3_^2-^] were generally low (often at or near the detection limit) with the highest concentrations found at Mine 1 (0.03 ± 0.05 mM; Table 1). S_2_O_3_^2-^ however, was detectable across all sites with a significantly higher (*p* < 0.001) average concentration at Mine 1 compared to the other three mines (Figure 3). [S_2_O_3_^2-^] was the largest measured S_React_ contributor, constituting 0 – 100% (calculated %S_2_O_3_^2-^ = [S_2_O_3_^2-^]/[S_React_] x 100) of the S_React_ pool at each mine. While Mine 4 had the largest average %S_React_, it had the smallest average %S_2_O_3_^2-^ (5.3 ± 8.2%) and therefore the largest proportion of unresolved sulfur species. On average, [S_2_O_3_^2-^] comprised approximately one-fifth of both Mine 1 (22 ± 16%) and Mine 2’s (19 ± 27%) [S_React_] pool.

### 3.3 TI wastewater physicochemistry

42 TI water cap samples were collected from the four mines between 2016 and 2019 during open water periods (early spring to late fall; Supplementary Figure S1) exhibited variable pH and DO values which are known to be important influencers of microbial ecological niches and associated sulfur oxidation pathways (Chen et al., 2013; Whaley-Martin et al., 2023). High temporal variability in DO (% saturation) and pH occurred for each mine as well as across mines, reflecting morphometric differences between TI facilities (particularly depth; Table 1) and the dynamic nature of actively managed TIs (Figure 3). Oxygen profiles from Mine 1, Mine 2, and Mine 3 demonstrated a steep oxygen gradient within TI water caps (Mine 4 profile data unavailable), ranging from <1 to >100% saturation (Table 1) across the four mines. Cross mine comparison revealed Mine 4 had significantly higher %DO compared to Mine 1 and Mine 2 (ANOVA and a post-hoc Tukey pairwise comparison test, *p* < 0.05; Figure 3) and no statistically significant differences among the remaining mines %DO.

Across the four mines, pH ranged from 5.1 to 11.8 (Table 1; Figure 3). Lime (Ca(OH)_2_) and alkaline tailings additions resulted in the highest average pH at Mine 1 (9.7 ± 2.0) and significantly higher pH values than Mine 2 (*p* < 0.001), Mine 3 (*p* < 0.05), and Mine 4 (*p* < 0.05) (Figure 3). Mine 2 TI waters had the lowest average pH (6.3 ± 0.9) with pH regularly falling below pH 6.5 (~64% of datapoints) and an observed decrease in pH from late spring to early fall. Mine 3’s TI water cap had the narrowest range of pH values (6.6 – 7.6, avg. = 7.1) with no observed seasonal fluctuations. Mine 4’s average pH was 8.0 ± 0.7 (7.2 – 9.0), with 75% of samples below pH 8.5, which was significantly higher than Mine 2 (*p* < 0.05) and lower than Mine 1 (*p* < 0.05; Figure 3). All four TIs exhibit dimictic lake mixing patterns, resulting in more homogenous dissolved oxygen and pH profiles during turnover events (spring and fall).

The acidity to SO_4_^2-^ ratio ([H^+^]/[SO_4_^2-^]) can be used as a proxy for discerning direct sulfur oxidation from disproportionation (Bernier and Warren, 2007; Whaley-Martin et al., 2023). Higher [H^+^]/[SO_4_^2-^] values observed at lower pH values indicate greater dominance of the c*sox* pathway, indicating more direct and complete oxidation to SO_4_^2-^ (higher acidity generation, minimal SOI generation; Supplementary Figure S3). Lower [H^+^]/[SO_4_^2-^] values observed at pH values >7, are consistent with more regeneration of SOI via disproportionation or partial oxidation attributed to i*sox*, r*dsr*, and S_4_I pathways activity, resulting in lower net acidity generation (Supplementary Figure S3). While these ratio values may be underestimates at pH values >7, where buffering capacity is likely present, the observed pH dependent [H^+^]/[SO_4_^2-^] trend is consistent with both S_2_O_3_^2-^ and SOB results. [S_2_O_3_^2-^] was positively correlated to pH (*p* < 0.0001, Pearson’s *r* = 0.80; Supplementary Figure S4A), while total TI SOB abundance was negatively correlated with pH (*p* < 0.01, Pearson’s *r* = −0.64; Supplementary Figure S4B). Interestingly, average mining and anthropogenic (M&A) literature and environmental literature values were consistent with the pattern observed here (Supplementary Figure S4B).

### 3.4 pH effect on SOB community structure

**Figure 4:**
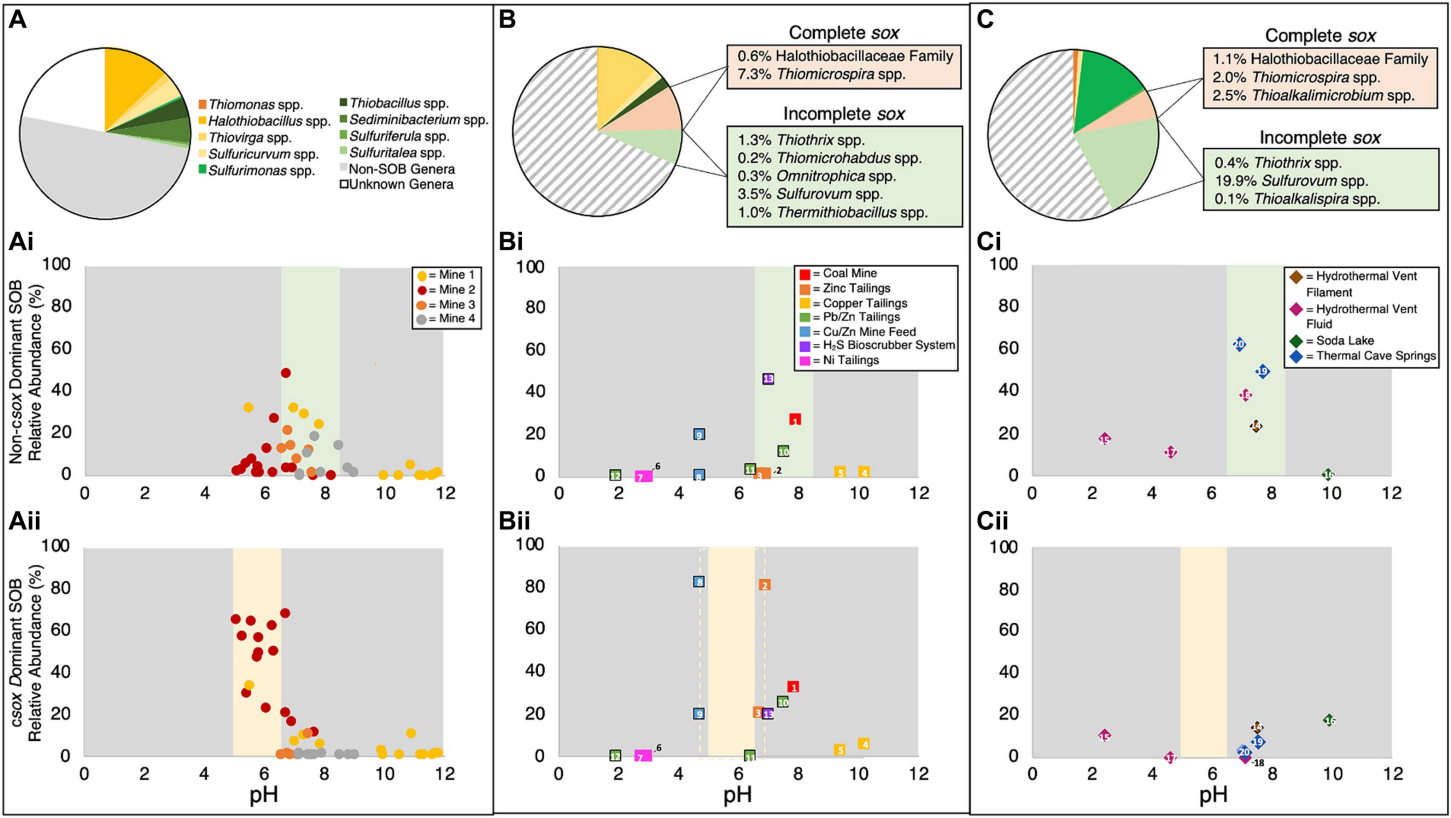
Genus level identification of **(A–C)** c*sox* dominant SOB relative abundances, **(Ai, Bi, Ci)** non-c*sox* dominant SOB relative abundances and **(Aii, Bii, Cii)** average 16S rRNA SOB communities identified in **(A, Ai, Aii)** tailings impoundment water samples from this study, **(B, Bi, Bii)** other mining and anthropogenic sample data from the literature and **(C, Ci, Cii)** environmental sample data obtained from the literature. “Non-SOB genera” are all other identified sequences in the samples and “unknown genera” are identified sequences not matched to genera in the Silva Database v138.1 (Data included in **Bi, Bii, Ci**, and **Cii** was collected from the following papers and can be identified using the superscript number: ^1^Kadnikov et al., 2019, ^2-5^Miettinen et al., 2021, ^6-7^Auld et al., 2017, ^8-9^Lopes et al., 2020, ^10-12^Chen et al., 2013, ^13^Haosagul et al., 2020, ^14^Patwardhan et al., 2018, ^15^Arce-Rodríguez et al., 2019, ^16^Vavourakis et al., 2019, ^17-18^Meier et al., 2017, ^19-20^Reigstad et al., 2011). Black outlines indicate non-water samples (e.g., soil, rock, tailings, biofilm, etc.).

Further examination of the relationship between SOB and pH for these four mine TIs, highlighted a pH dependent pattern in the occurrence of c*sox* and non-c*sox* dominant SOB. While clear relationships between pH and total SOB abundance, [S_2_O_3_^2-^], and [H^+^]/[SO_4_^2-^] ratios were observed, no discernible correlation was identified between dissolved oxygen and any of these parameters. Peak abundances of non-c*sox* dominant SOB were observed at circumneutral pH (pH ~6 to ~8.5; Figure 4Ai) while the highest abundances of c*sox* dominant SOB were observed below pH ~6.5 (Figure 4Aii). Samples above pH ~8.5 had low abundances of both c*sox* and non-c*sox* dominant SOB (Figures 4Ai,ii). Current literature indicates at least two genera of SOB (*Thioalkalimicrobium* and *Thioalkalivibrio*) are capable of growth under alkaline conditions (Sorokin et al., 2001) though neither were observed in this study’s TI samples. Lower pH value (pH < ~6.5) TI waters had the highest abundances of c*sox* dominant SOB, lower [S_2_O_3_^2-^] and higher [H^+^]/[SO_4_^2-^] ratios; collectively consistent with direct oxidation via the c*sox* pathway (Figure 4Aii). At more circumneutral pH values (pH ~6 to ~8.5), where non-c*sox* dominant SOB dominated, higher [S_2_O_3_^2-^] and low [H^+^]/[SO_4_^2-^] values were also observed, indicating distinct pH dependent SOB community structure and S pathway(s) (Figures 4Ai,ii).

#### 3.4.1 Comparison to broader environments

The exploration of the pH – c*sox* dominant/non-c*sox* dominant SOB relationship extended across diverse contexts by integrating literature data (pH and SOB abundance) from broader mining environments, an industrial H_2_S bioscrubber, and a variety of natural environmental sites (e.g., hydrothermal vents, soda lakes, and thermal cave springs) (Reigstad et al., 2011; Meier et al., 2017; Patwardhan et al., 2018; Arce-Rodríguez et al., 2019; Vavourakis et al., 2019) (see Supplementary Table S1). Genus level SOB abundances were categorized into c*sox* dominant SOB and non-c*sox* dominant SOB. Literature identified SOB (not found in this study’s TI samples) were classified as c*sox* dominant and non-c*sox* dominant SOB using available genetic data and consensus of the current literature based on the reported presence of a complete or incomplete *sox* pathway. Additional SOB genera included three c*sox* dominant clades *Thiomicrospira* spp., *Thioalkalimicrobium* spp., and *Halothiobacilleaceae* Family and seven non-c*sox* dominant clades (*Thiothrix* spp., *Thiomicrohabdus* spp., *Omnitrophica* spp., *Sulfurovum* spp., *Thermithiobacillus* spp., *Sulfobacillus* spp., and *Thioalkalispira* spp.). Though class and family level abundances have been published for TI water cap samples, genus level data were not widely available in the literature. Data from various alternative sample locations (e.g., water from mill feed, solid tailings pore water), including both solid and water samples, were used. This approach aimed to broaden the range of geochemical and physicochemical characteristic investigated.

A brief summary and comparison of the relevant data for the overall groups of samples are provided in Supplementary Table S1 as more complete descriptions for individual sites can be found in their respective publications. The collective M&A literature data (*n* = 13) covered a pH range from 1.9 to 10.2 (Supplementary Table S1), the environmental samples (*n* = 7) ranged from pH 2.4 to 9.9 (Supplementary Table S1), while this study’s TI samples ranged from pH 5.1 to 11.8 (Table 1). Average total SOB abundance of this study’s TI samples (29 ± 26%) and M&A literature samples (31 ± 31%) were similar, while the average total SOB abundance for the environmental literature samples (42 ± 26%) was >10% higher (Figures 4A–C). Additional SOB genera (outside of the nine identified for TI in this study) represented ~15% (or ~ 50% of the total SOB) of the M&A literature and ~ 26% (or ~ 62% of the total SOB) of the environmental literature samples (Figures 4B,C). Both the M&A and environmental literature SOB communities diverged by at least 50% from those characterized here for base metal TI wastewaters, indicating notable disparities in microbial community composition suggesting SOB genera specific ecological niches.

On average, this study’s samples had ~64% of the total SOB abundance consisting of c*sox* dominant SOB, while ~36% were classified as non-c*sox* dominant (Figure 4A). The M&A literature samples averaged slightly higher c*sox* dominant SOB contribution with ~72% of the total SOB abundance being attributed to c*sox* dominant SOB (Figure 4B). Conversely, the environmental literature samples averaged a much lower contribution of c*sox* dominant SOB, contributing only ~18% of the total SOB abundance (Figure 4C). *Thiovirga* spp. and *Thiomicrospira* spp., both classified as c*sox* dominant SOB, were the two most abundant SOB in the M&A literature samples, while the two most abundant SOB in the environmental samples were both classified as non-c*sox* dominant SOB (*Sulfurimonas* spp. and *Sulfurovum* spp.) (Figures 4B,C; Supplementary Table S1). In contrast, the two most abundant SOB from this study’s TI waters were *Halothiobacillus* spp. (c*sox* dominant) and *Sediminibacterium* spp. (non-c*sox* dominant). *Thiomicrohabdus* spp., *Omnitrophica* spp., and *Thermithiobacillus* spp. were unique to M&A samples with *Thiomicrohabdus* spp. and *Omnitrophica* spp. only being found in a single sample (Kadnikov et al., 2019) and *Thermithiobacillus* spp. identified in two samples from the same mine (Lopes et al., 2020). *Thioalkalispira* spp. and *Thioalkalimicrobium* spp. were unique to one environmental sample (Vavourakis et al., 2019).

Four M&A literature samples were found to have elevated abundances (>10%) of non-c*sox* dominant SOB including three solid samples and one water sample (Figure 4Bii). Non-c*sox* dominant SOB abundances of the M&A literature samples were found to be highest in the only non-mining sample (13 SPM Swine Farm H_2_S Bioscrubber System; Figure 4Bii). Three of the four M&A literature samples with elevated SOB abundances fell between pH ~6.5 and ~ 8, covering a similar range to the samples included in this study (Figure 4Bi). Among the seven M&A literature samples displaying elevated (>10%) c*sox* dominant SOB abundances, five were found to occur within a pH range of ~5 and ~ 7 (Figure 4Bii); a slightly higher upper range compared to this study’s TI results (< pH 6.5; Figure 4Aii). The highest c*sox* dominant SOB abundances in M&A literature samples were identified in samples at pH ~5 and ~ 7 (Figure 4Bii) though no data between those two pH values are presently available in the literature. At pH >8, there were two M&A literature samples which both showed low abundances of both c*sox* dominant and non-c*sox* dominant SOB (Figures 4Bi,ii), aligned with the pattern of low SOB abundance at more alkaline pH observed in this study’s TI samples. Samples below pH 4 (closer to acid mine drainage conditions) from the M&A literature samples had very low SOB abundances (Figures 4Bi,ii) in both solid and water samples.

Differences observed between the M&A literature data and this study’s TI data may reflect differences in S substrate availability due to the types or locations of samples included. The M&A mining-related solid sample’s primary source of sulfur would be sulfide-containing ores whereas SOI, aqueous dissolved species, prevalent in the TI waters investigated here, would be limited in solid samples. Solid tailings and feed samples may similarly exhibit different patterns of SOB community structure and abundance and associated sulfur oxidation genes due to these differences in available sulfur species, as only one of the three main pathways highlighted here (*sox*) utilizes sulfide (Klatt and Polerecky, 2015). This may account for the elevated c*sox* dominant SOB abundances observed in some of the circumneutral solid samples included in Figure 4Bii. Extrapolating the pattern identified in this study’s samples to other anthropogenically impacted environments would require more data (both genus level abundance and pH data) for TI water caps which are not currently available. However, these comparative results, across a range of systems where data are available, show some consistencies in patterns with those observed for base metal TI wastewaters, namely low SOB abundances at elevated pH values (pH >8) and similar pH ranges for the peak functional pathway abundances of both non-c*sox* dominant (pH ~6.5 to ~8.5) and c*sox* dominant (~4.5 to ~7) SOB (Figures 4Bi,ii).

The environmental literature samples that fell between pH ~6.5 and ~ 8.5 had elevated non-c*sox* dominant SOB abundances (Figure 4Ci) consistent with results for this study’s TI waters. No environmental literature samples were found between pH ~5 and ~ 6.5 precluding a direct comparison to the peak c*sox* dominant SOB abundances observed for TI here. Similar to the observed TI results here, however, three environmental literature samples (two samples <pH ~5 and one sample > pH ~8.5) had lower abundances of non-c*sox* dominant SOB than those within the more circum-neutral range (pH ~6.5 – ~8.5) (Figure 4Ci). The environmental literature group of samples was the only group where all samples had >1% non-c*sox* dominant SOB (Figure 4Ci). Unlike the previous two groups of samples, none of the environmental samples had high abundances of c*sox* dominant SOB (all <20% c*sox* dominant SOB abundance; Figure 4Cii). This may reflect typically much lower environmental SOI concentrations (O’Brien and Birkner, 1977) and therefore metabolisms that exhaust SOI by completely oxidizing them to SO_4_^2-^ (i.e., c*sox*) may not be favoured over those that disproportionate or recycle SOI such as the S_4_I or r*dsr* pathways (ie. non-c*sox* dominant SOB). The divergence of mining impacted environments (both in this study (oxic TI wastewaters) and literature data) from natural environments suggests a specialized mining specific microbiome with higher abundance(s) of c*sox* dominant SOB favoured by higher concentrations of S species in these contexts.

### 3.5 Factors influencing microbial community structure and function

The i*sox* pathway or the i*sox* + r*dsr* pathway generates more ATP and is more efficient due to energy conservation via S^0^ production and storage (Klatt and Polerecky, 2015). While energy efficiency is important, it is not the sole determinant of success in any given environment (Klatt and Polerecky, 2015). This is exemplified across the diverse set of environments presented here, where both the i*sox* or i*sox* + *rdsr* pathway, as well as the c*sox* pathway appear at different times. Microbial success can be determined by growth rate, which considers both the substrate uptake rate and the growth yield (Klatt and Polerecky, 2015). Both growth yield and efficiency are directly related, but lower efficiency (such as that associated with c*sox*) can be made up for by speed, resulting in equal or greater success (Sorokin and Kuenen, 2005; Klatt and Polerecky, 2015). Whaley-Martin et al. (2023) provided laboratory enrichment data from the same four mines included in this study to showcase that c*sox* SOB (*Halothiobacillus* spp. dominant) enrichments exhibit a considerably faster S_2_O_3_^2-^ oxidation rate compared to the enrichments with r*dsr* containing SOB. Growth strategies which rely on speed are only likely to dominate in environments with unlimited substrates (Klatt and Polerecky, 2015). Though mining and other industrial environments technically do not have unlimited SOI, there are consistent replenishments of SOI through tailings additions or continuous wastewater treatment/flow. The observed proliferation of SOB with the c*sox* pathway in mining and industrial contexts (Figures 4Aii,Bii), aligns with their demonstrated capability for rapid S_2_O_3_^2-^ oxidation under primarily oxic conditions. This highlights their competitive advantage in these substrate-rich environments where consistent SOI inputs help sustain their growth. The transition from non-c*sox* dominant SOB (i*sox* or i*sox* + r*dsr*) to c*sox* dominant SOB occurs within the range of 0.1 to 0.3 mmol/L [S_2_O_3_^2-^] (Supplementary Figure S4A), suggesting a potential SOI threshold for the initiation of this metabolic shift. The associated pH decrease with the increased presence of c*sox* dominant SOB may occur as a by-product of the activity of the c*sox* pathway which produces SO_4_^2-^ and acidity and may slowly decrease pH. Further, under natural environmental conditions, where SOI are not as readily replenished, we do not see the proliferation of the c*sox* pathway (Figure 4Cii).

**Figure 5:**
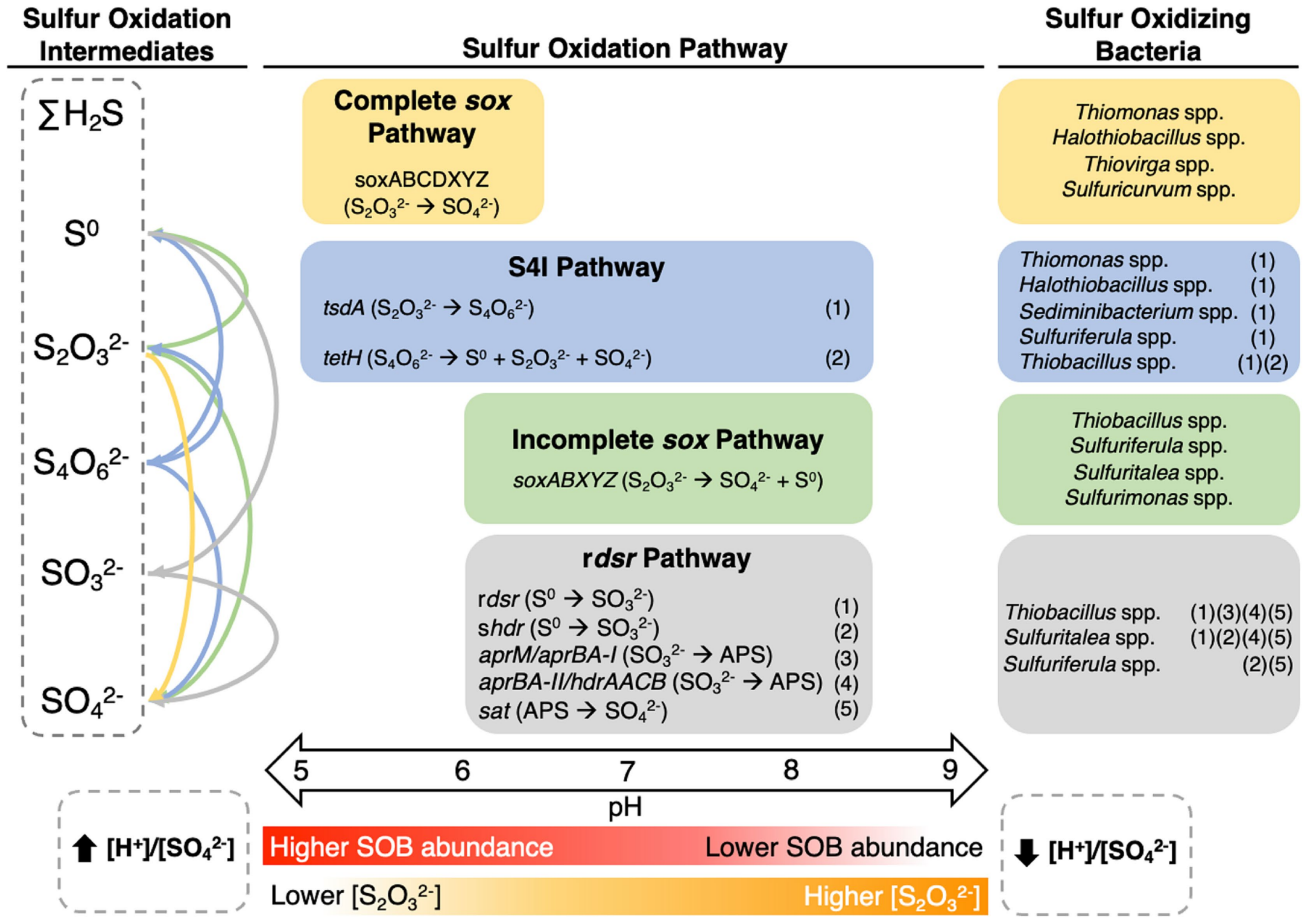
Conceptual model of pH bound metabolic activity with the associated responsible genera and geochemical pathway and outcomes in metal mining TI waters.

Across the four TIs investigated here, evidence of pH and [S_2_O_3_^2-^] dependent occurrence of all four key metabolic pathways, c*sox* pathway, S_4_I pathway, i*sox* pathway, and r*dsr* pathway emerged (Figure 5). pH partitioned three of these pathways: namely c*sox* pathway dominated at lower pH values (pH ~5 to ~6.5) while the i*sox* and r*dsr* pathways were more prevalent at circumneutral pH values (pH ~6.5 to ~8.5; Figure 5). The shift from circumneutral to more acidic pH may represent the onset of a positive feedback loop led by c*sox* dominant SOB such as *Halothiobacillus* spp. which has previously been implicated in catalyzing the shift to net acid generation in laboratory scale experiments (Whaley-Martin et al., 2019). The c*sox* pathway, responsible for the direct oxidation of S_2_O_3_^2-^ to SO_4_^2-^, which generates more acidity than the S_4_I, i*sox*, or r*dsr* pathway results in decreasing [S_2_O_3_^2-^] and increasing [H^+^]/[SO_4_^2-^] values (Figure 5). Uniquely, portions of the S_4_I pathway, namely the *tsdA* gene, were observed in SOB genera from across the entire pH range (Figure 5).

## 4 Conclusion

Results here reveal new insights into the interplay between pH, SOB community composition and function (c*sox* dominant vs. non-c*sox* dominant), S cycling and acidity generation within primarily oxic mine TI waters over seasonal, annual, and mine operation scales. Our results highlight two key pH dependent niches characterized by the presence of either (i) c*sox* dominant SOB (e.g., *Thiomonas* spp., *Halothiobacillus* spp.) associated with lower pH values and lower [S_2_O_3_^2-^] or (ii) non-c*sox* dominant SOB (i*sox* and/or r*dsr* pathways; e.g. *Thiobacillus* spp., *Sulfuriferula* spp.) associated with more circumneutral pH conditions and higher [S_2_O_3_^2-^] (Figure 5). The presence of the first part of the S_4_I pathway (*tsdA*; S_2_O_3_^2-^ to S_4_O_6_^2-^) was ubiquitous across pH and [S_2_O_3_^2-^] niches; while possible subsequent processing of S_4_O_6_^2-^ via *tetH* which can lead to S^0^, and S_2_O_3_^2-^ regeneration, as well as SO_4_^2-^, was limited to *Thiobacillus* spp., observed more prevalently in circumneutral pH values (Figure 5). Extrapolation of these results to broader environments via comparative analysis using M&A and environmental literature data revealed a specialized mining SOB microbiome characterized by elevated abundances of c*sox* dominant SOB. Elevated SOI concentrations, typical of mining environments, support the establishment and sustainment of c*sox* dominant communities unique to these environments. Our comprehensive study into SOB community dynamics, sulfur oxidation pathways, and the influence of geochemical and physicochemical factors in mining impacted waters, highlight the importance of thiosulfate availability, and pH constraints on sulfur oxidizing metabolism under oxic conditions, prevalent in TI contexts. This study highlights opportunities to manipulate TI SOB communities through pH adjustment and/or [S_2_O_3_^2-^] management, offering potential avenues to reduce the risk of SOI discharge into receiving waters.

## Data availability statement

The 16S rRNA sequencing data generated from this study have been deposited in the NCBI database under accession codes PRJNA510600, PRJNA882497, and PRJNA930015. The metagenome-assembled genomes generated in this study are available at ggKbase.berkeley.edu via https://ggkbase.berkeley.edu/SOB_across_mines/organisms.

## Author contributions

LT: Data curation, Formal analysis, Investigation, Methodology, Visualization, Writing – original draft, Writing – review & editing, Resources, Software. KW-M: Investigation, Methodology, Project administration, Writing – review & editing. L-XC: Data curation, Formal analysis, Software, Writing – review & editing. TC: Investigation, Methodology, Project administration, Supervision, Writing – review & editing. JA: Formal analysis, Investigation, Writing – original draft, Writing – review & editing. CJ: Methodology, Resources, Writing – review & editing. JK: Methodology, Resources, Writing – review & editing. LR: Methodology, Resources, Writing – review & editing. HS: Methodology, Resources, Writing – review & editing. JB: Conceptualization, Funding acquisition, Resources, Writing – review & editing. SA: Resources, Writing – review & editing, Methodology. LW: Conceptualization, Funding acquisition, Project administration, Resources, Supervision, Writing – review & editing.

## Footnotes

^1^ggkbase.berkeley.edu
