# Supplementary material for: pH and thiosulfate dependent microbial sulfur oxidation strategies across diverse environments
Source: Front Microbiol. 2024 Jul 19;15:1426584. doi: 10.3389/fmicb.2024.1426584 (PMC11294248; doi:10.3389/fmicb.2024.1426584)
Supplement: Supplementary file 1 [file Data_Sheet_1.docx]

Supplementary Material

pH and Thiosulfate Dependent Microbial Sulfur Oxidation Strategies across Diverse Environments

**Lauren E. Twible^1^, Kelly Whaley-Martin^1^, Lin-Xing Chen^2^, Tara Colenbrander Nelson^1^, James L.S. Arrey^1^, Chad V. Jarolimek^3^, Josh J. King^4^, Lisa Ramilo^5^, Helga Sonnenberg^6^, Jillian F. Banfield^2^, Simon C. Apte^7^, and Lesley A. Warren^1*^**

*** Correspondence:**Dr. Lesley A. Warren

[lesley.warren@utoronto.ca](mailto:lesley.warren@utoronto.ca)

**Supplementary Figure 1:** Number of samples included in study for each mine, year, and month (the number of samples taken at each time point are included in the corresponding circle).

**Supplementary Figure 2:** Outlines of the four tailings impoundments included in this study and sampling locations ( ). Scale approximated using Google Maps 2022.

**Supplementary Figure 3:** Ratios of [H^+^]/[SO_4_^2-^] for all 42 samples in order of ascending pH.

**Supplementary Figure 4:** Linear regression between **(A)** pH and [S_2_O_3_^2-^] (y = 0.15x - 0.89, Pearson’s r = 0.80) and **(B)** pH and total SOB abundance (%) (y = -8.56x + 94.6, Pearson’s r = -0.64) across all 42 samples (2016 – 2019), presented with 95% confidence and 95% prediction bands (p < 0.01). Average total SOB abundances for the mining and anthropogenic literature samples ( ) and environmental literature samples ( ) were also included.

**Supplementary Table 1:** Literature sample information including classification summaries and pH.

| Sample ID | Sample Type | Location | pH | c*sox* Dominant SOB Abundance (%) | Non-cs*ox* Dominant SOB Abundance (%) | Total SOB Abundance (%) | Reference |
| --- | --- | --- | --- | --- | --- | --- | --- |
| Flooded Coal Mine Shaft | Water | Russia | 7.87 | 33 | 27 | 60 | Kadnikov et al., 2019 |
| Zinc Tailings (Summer) | Water | Portugal | 6.9 | 81 | 1 | 82 | Miettinen et al., 2021 |
| Zinc Tailings (Winter) | Water | Portugal | 6.7 | 21 | 1 | 22 | Miettinen et al., 2021 |
| Copper Tailings (Summer) | Water | Portugal | 10.2 | 6 | 2 | 8 | Miettinen et al., 2021 |
| Copper Tailings (Winter) | Water | Portugal | 9.4 | 3 | 2 | 5 | Miettinen et al., 2021 |
| Vale Copper Cliff Nickel AMD Tailings (Winter) | Water | Canada | 2.94 | 0 | 0 | 0 | Auld et al., 2017 |
| Vale Copper Cliff Nickel AMD Tailings (Summer) | Water | Canada | 2.75 | 0 | 0 | 0 | Auld et al., 2017 |
| Neves Corvo Mine Mill Feed (Summer) | Dry Ore Fragments | Portugal | 4.7 | 82 | <1 | 83 | Lopes et al., 2020 |
| Neves Corvo Mine Mill Feed (Spring) | Dry Ore Fragments | Portugal | 4.7 | 21 | 20 | 41 | Lopes et al., 2020 |
| Fankou Pb/Zn Mine Primary Tailings | Dried Tailings | China | 7.5 | 26 | 12 | 38 | Chen et al., 2013 |
| Fankou Pb/Zn Mine Primary Tailings (“Slightly Acidic”) | Dried Tailings | China | 6.4 | <1 | 3 | 3 | Chen et al., 2013 |
| Fankou Pb/Zn Mine Primary Tailings (“Acidic”) | Dried Tailings | China | 1.9 | <1 | <1 | 1 | Chen et al., 2013 |
| SPM Swine Farm H_2_S Bioscrubber System | Microbial Sludge | Thailand | 7 | 20 | 47 | 67 | Haosagul et al., 2020 |
| Tor Caldara Sea Vent Filament | Vent Filament | Italy | 7.48 | 14 | 24 | 38 | Patwardhan et al., 2018 |
| Wet Mofette Hydrothermal Vent | Water | Costa Rica | 2.4 | 10 | 18 | 28 | Arce-Rodríguez et al., 2019 |
| Cock Soda Lake Brine | Water | Russia | 9.9 | 18 | 1 | 19 | Vavourakis et al., 2019 |
| North Susu Knolls Hydrothermal Field (1154m) | Water | Papua New Guinea | 4.6 | 0 | 12 | 12 | Meier et al., 2017 |
| Roman Ruins PACManus Hydrothermal Field (1685m) | Water | Papua New Guinea | 7.1 | 0 | 39 | 39 | Meier et al., 2017 |
| Fisosen Thermal Spring | Water | Norway | 7.5 | 8 | 76 | 84 | Reigstad et al., 2011 |
| Trollosen Thermal Spring | Water | Norway | 7 | 3 | 74 | 77 | Reigstad et al., 2011 |

**Supplementary Table 2**: 116 Genomes reconstructed from 38 samples collected between 2016 and 2018 from all 4 mine tailings impoundment waters.

| Sample Location | Sample Date (mm/dd/yyyy) | Genus ID |
| --- | --- | --- |
| Mine 2 | 11/07/2017 | Sulfuriferula |
| Mine 4 | 01/24/2018 |  |
| Mine 4 | 02/27/2018 |  |
| Mine 4 | 03/22/2018 |  |
| Mine 4 | 05/23/2018 |  |
| Mine 4 | 11/22/2018 |  |
| Mine 4 | 11/25/2017 |  |
| Mine 1 | 07/26/2016 | Sulfuricurvum |
| Mine 2 | 07/10/2018 |  |
| Mine 2 | 07/24/2016 |  |
| Mine 2 | 07/24/2016 |  |
| Mine 3 | 09/25/2017 | Halothiobacillus |
| Mine 1 | 07/26/2016 |  |
| Mine 1 | 09/25/2017 |  |
| Mine 1 | 09/25/2017 |  |
| Mine 1 | 09/25/2017 |  |
| Mine 1 | 09/25/2017 |  |
| Mine 2 | 05/31/2017 |  |
| Mine 2 | 06/10/2018 |  |
| Mine 2 | 06/27/2017 |  |
| Mine 2 | 06/27/2017 |  |
| Mine 2 | 07/10/2018 |  |
| Mine 2 | 07/11/2018 |  |
| Mine 2 | 07/11/2018 |  |
| Mine 2 | 07/18/2017 |  |
| Mine 2 | 07/18/2017 |  |
| Mine 2 | 07/18/2017 |  |
| Mine 2 | 07/23/2018 |  |
| Mine 2 | 07/24/2016 |  |
| Mine 2 | 07/24/2016 |  |
| Mine 2 | 07/24/2016 |  |
| Mine 2 | 08/08/2017 |  |
| Mine 2 | 08/08/2017 |  |
| Mine 2 | 08/08/2017 |  |
| Mine 2 | 08/14/2018 |  |
| Mine 2 | 08/14/2018 |  |
| Mine 2 | 08/14/2018 |  |
| Mine 2 | 09/20/2016 |  |
| Mine 2 | 09/27/2017 |  |
| Mine 2 | 09/27/2017 |  |
| Mine 2 | 11/07/2017 |  |
| Mine 2 | 11/07/2017 |  |
| Mine 3 | 03/12/2018 | Sediminibacterium |
| Mine 3 | 09/25/2017 |  |
| Mine 1 | 07/xx/2018 |  |
| Mine 1 | 07/xx/2018 |  |
| Mine 1 | 09/24/2018 |  |
| Mine 1 | 09/24/2018 |  |
| Mine 1 | 09/25/2017 |  |
| Mine 1 | 09/25/2017 |  |
| Mine 2 | 07/10/2018 |  |
| Mine 2 | 07/11/2018 |  |
| Mine 2 | 07/18/2017 |  |
| Mine 2 | 07/11/2018 |  |
| Mine 2 | 07/18/2017 |  |
| Mine 2 | 07/23/2018 |  |
| Mine 2 | 07/24/2016 |  |
| Mine 2 | 07/24/2016 |  |
| Mine 2 | 08/08/2017 |  |
| Mine 2 | 08/08/2017 |  |
| Mine 2 | 08/14/2018 |  |
| Mine 2 | 08/14/2018 |  |
| Mine 2 | 08/14/2018 |  |
| Mine 2 | 08/14/2018 |  |
| Mine 2 | 09/20/2016 |  |
| Mine 2 | 09/27/2017 |  |
| Mine 2 | 10/15/2018 |  |
| Mine 2 | 10/15/2018 |  |
| Mine 4 | 07/25/2018 |  |
| Mine 4 | 08/xx/2018 |  |
| Mine 4 | 08/28/2017 |  |
| Mine 4 | 09/25/2017 |  |
| Mine 1 | 07/26/2016 | Thiomonas |
| Mine 2 | 07/10/2018 |  |
| Mine 2 | 07/23/2018 |  |
| Mine 3 | 09/25/2017 | Thiobacillus |
| Mine 3 | 09/25/2017 |  |
| Mine 3 | 09/25/2017 |  |
| Mine 1 | 06/xx/2018 |  |
| Mine 1 | 07/xx/2018 |  |
| Mine 1 | 07/xx/2018 |  |
| Mine 1 | 07/26/2016 |  |
| Mine 1 | 09/24/2018 |  |
| Mine 2 | 07/11/2018 |  |
| Mine 2 | 08/14/2018 |  |
| Mine 2 | 08/14/2018 |  |
| Mine 2 | 08/14/2018 |  |
| Mine 2 | 08/14/2018 |  |
| Mine 2 | 08/14/2018 |  |
| Mine 2 | 08/14/2018 |  |
| Mine 2 | 08/14/2018 |  |
| Mine 4 | 01/24/2018 |  |
| Mine 4 | 02/27/2018 |  |
| Mine 4 | 02/27/2018 |  |
| Mine 4 | 02/27/2018 |  |
| Mine 4 | 03/22/2018 |  |
| Mine 4 | 03/22/2018 |  |
| Mine 4 | 07/25/2018 |  |
| Mine 4 | 08/xx/2018 |  |
| Mine 4 | 08/xx/2018 |  |
| Mine 4 | 09/xx/2018 |  |
| Mine 4 | 09/xx/2018 |  |
| Mine 4 | 09/25/2017 |  |
| Mine 4 | 10/24/2017 |  |
| Mine 4 | 10/24/2017 |  |
| Mine 4 | 11/22/2018 |  |
| Mine 2 | 08/14/2018 | Thiovirga |
| Mine 2 | 08/14/2018 |  |
| Mine 2 | 08/14/2018 |  |

**
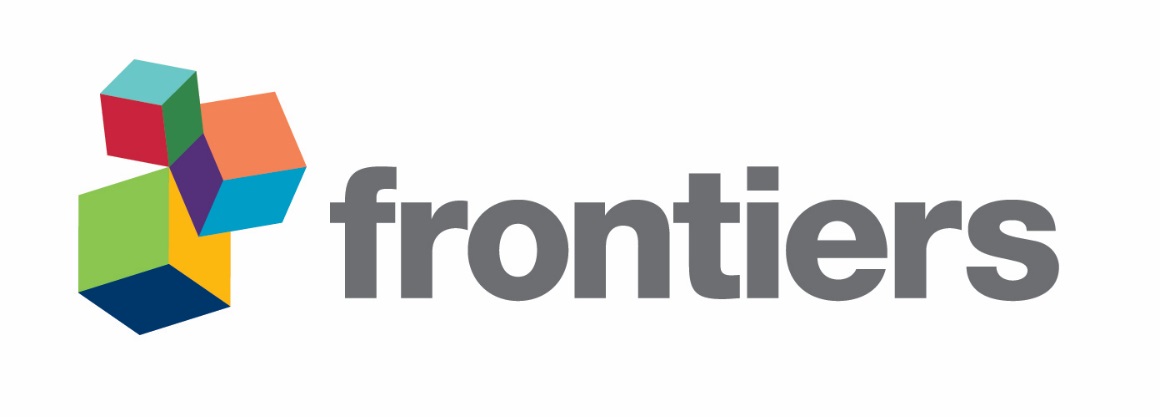
**
